# Identifying Weak Signals in Inhomogeneous Neuronal Images for Large-Scale Tracing of Sparsely Distributed Neurites

**DOI:** 10.1007/s12021-018-9414-9

**Published:** 2019-01-11

**Authors:** Shiwei Li, Tingwei Quan, Hang Zhou, FangFang Yin, Anan Li, Ling Fu, Qingming Luo, Hui Gong, Shaoqun Zeng

**Affiliations:** 1 Britton Chance Center for Biomedical Photonics, Wuhan National Laboratory for Optoelectronics-Huazhong University of Science and Technology, Wuhan, 430074 Hubei China; 2 MOE Key Laboratory for Biomedical Photonics, Collaborative Innovation Center for Biomedical Engineering, School of Engineering Sciences, Huazhong University of Science and Technology, Wuhan, 430074 Hubei China; 3 School of Mathematics and Economics, Hubei University of Education, Wuhan, 430205 Hubei China

**Keywords:** Neuron tracing, Weak signal identification, Large-scale neuronal reconstruction, Support vector machine

## Abstract

Tracing neurites constitutes the core of neuronal morphology reconstruction, a key step toward neuronal circuit mapping. Modern optical-imaging techniques allow observation of nearly complete mouse neuron morphologies across brain regions or even the whole brain. However, high-level automation reconstruction of neurons, i.e., the reconstruction with a few of manual edits requires discrimination of weak foreground points from the inhomogeneous background. We constructed an identification model, where empirical observations made from neuronal images were summarized into rules for designing feature vectors that to classify foreground and background, and a support vector machine (SVM) was used to learn these feature vectors. We embedded this constructed SVM classifier into a previously developed tool, SparseTracer, to obtain SparseTracer-Learned Feature Vector (ST-LFV). ST-LFV can trace sparsely distributed neurites with weak signals (contrast-to-noise ratio < 1.5) against an inhomogeneous background in datasets imaged by widely used light-microscopy techniques like confocal microscopy and two-photon microscopy. Moreover, 12 sub-blocks were extracted from different brain regions*.* The average recall and precision rates were 99% and 97%, respectively. These results indicated that ST-LFV is well suited for weak signal identification with varying image characteristics. We also applied ST-LFV to trace long-range neurites from images where neurites are sparsely distributed but their image intensities are weak in some cases*.* When tracing this long-range neurites, manual edit was required once to obtain results equivalent to the ground truth, compared with 20 times of manual edits required by SparseTracer. This improvement in the level of automatic reconstruction indicates that ST-LFV has the potential to rapidly reconstruct sparsely distributed neurons at the large scale.

**Electronic supplementary material:**

The online version of this article (10.1007/s12021-018-9414-9) contains supplementary material, which is available to authorized users.

## Introduction

Structural and functional mapping of neuronal circuits is one of the central tasks in neuroanatomical studies (Mitra 2014; Osten and Margrie 2013). Mapping the neuronal circuit largely depends on reconstructing the morphologies of neurons (Parekh and Ascoli 2013; Donohue and Ascoli 2011; Meijering 2010; Svoboda 2011), which are usually considered as the basic structural unit in the circuit (Marx 2012). Neurites form the core of neuronal morphologies (Parekh and Ascoli 2015; Peng et al. 2015), hence, tracing neurites plays an important role in neuronal morphology reconstruction.

In recent years, a series of breakthroughs in molecular labeling (Feng et al. 2000; Jefferis and Livet 2012; Luo and Callaway 2008; Ugolini 2010) and optical imaging techniques (Chung and Deisseroth 2013; Gong et al. 2013; Gong et al. 2016; Osten and Margrie 2013; Ragan et al. 2012; Silvestri et al. 2012) have enabled the rapid collection of brain-wide neuronal images at submicron resolutions. These techniques have been used to map the neuronal circuit of mice (Osten and Margrie 2013; Fürth et al. 2018). However, automatic tracing methods are error-prone and may fail in neuronal structures with weak signal intensity against an inhomogeneous background. These structures hamper the accuracy of overall neuronal reconstruction. In addition, tracing the circuit from tens of thousands of images is laborious (Marx 2012; Zingg et al. 2014).

Generally, the above challenges originate from the optical imaging strategy and complicated nature of neuronal morphologies. First, whole brain imaging is usually implemented at a relatively low spatial sampling rate to achieve a balance between the sampling rate and the imaging speed. Second, neurites with small radii (several hundred nanometers) contain few fluorescent molecules. These facts contribute to the presence of neurites with low signal intensity after fluorescent imaging. Third, long-term imaging procedures and structural differences in different brain regions result in an inhomogeneous background, which increase the difficulty in identifying weak signals.

**Fig. 1:**
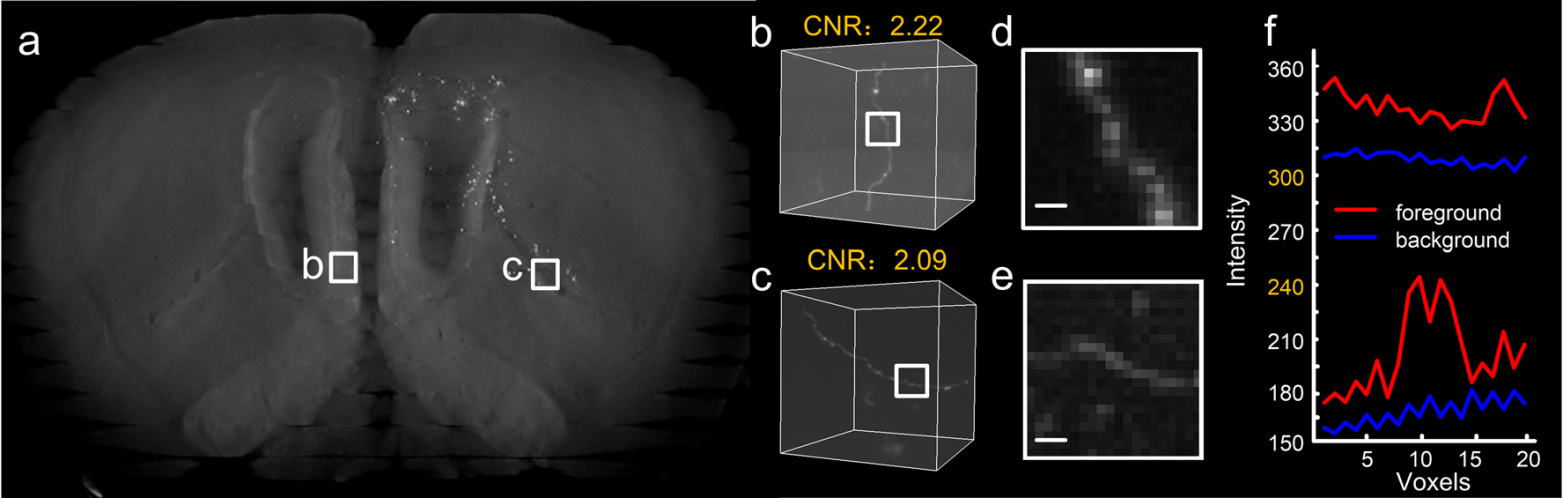
Some key characteristics of neuronal images at the brain-wide scale. **a** A thumbnail view of a mouse brain dataset in which two sub-blocks are highlighted with squares; **b** a sub-block with a single neurite, part of which is labeled with a square; **c** similar to (b); **d** maximum projections of the labeled view in (b) through a depth of 10 μm, with a scale bar of 2 μm; **e** similar to (d); **f** the upper two curves represent the foreground (red) and the background (blue) of the neurite in (d), and the bottom two curves correspond to the neurite in (e)

Some key characteristics of neuronal images are illustrated in Fig. 1. Two sub-blocks were extracted from a whole-brain imaging dataset (Fig. 1a-c), and one sub-block (Fig. 1c) contains a neurite with a contrast-to-noise ratio (Song et al. 2004) as low as 2.09 (see Supplementary note). Furthermore, when computing the foreground and background profiles of a portion of two sub-blocks (Fig. 1d, e), the background intensity of sub-block *b* is even higher than the foreground intensity of sub-block *c* (Fig. 1f), indicating the existence of an inhomogeneous background*.*

Many methods exhibit great neurite tracing performance and demonstrate a good ability to identify neurites with weak signals, such as, the model-fitting (Zhao et al. 2011; Santamaria-Pang et al. 2015), open-snake (Wang et al. 2011; Cai et al. 2006; Luo et al. 2015; Xu and Prince 1998), graph-based (Peng et al. 2010; Turetken et al. 2011; Basu et al. 2013; Chothani et al. 2011; Yang et al. 2013), principal curve (Bas and Erdogmus 2011), voxel scooping (Rodriguez et al. 2009), multi-scale tracking (Choromanska et al. 2012; Frangi et al. 1988), and density filters (Radojevic and Meijering 2017) techniques. However, most of these methods require fine and frequent parameter tuning to trace neurites with weak signals. This may lead to difficulties in separating weak signals from an inhomogeneous background. Recent machine learning methods (Li et al. 2017; Chen et al. 2015; Megjhani et al. 2015; Gu et al. 2017; Hernandez-Herrera et al. 2016; Becker et al. 2013) can provide more accurate tracing results than traditional approaches. However, the largest reported volume size is limited to hundreds of megabytes and the corresponding computational cost ranges from several tens of minutes to several hours (Hernandez-Herrera et al. 2016; Li et al. 2017). This indicates that these methods may not be able to trace neurites rapidly in large-scale images without the help of GPU computing or distributed computing, which is attributed to two primary reasons. First, machine learning methods consider many image features and result in a complicated framework, thereby requiring intensive computations. Second*,* some methods separate the procedures of identifying foreground voxels and tracing the voxels into neurite skeletons (Li et al. 2017; Chen et al. 2015; Megjhani et al. 2015; Gu et al. 2017; Hernandez-Herrera et al. 2016; Becker et al. 2013). These techniques attempt to identify as many foreground voxels as possible, which generates the detailed shape of a neurite. Thus, larger images incur heavier computational costs.

In this study, we propose a method for identifying weak signals and embed this method into the neurite tracing pipeline. Our strategy closely links the identification and tracing procedures and requires only a few foreground voxels in the tracing process for identification. We observed many neuronal images and determined identification rules: the local background is smooth, the neurite has a strong anisotropic shape, and the difference in image intensities between the neurite and its local background makes them separable. These rules are applicable to several neuronal images but may not hold in all cases. Applying preprocessing techniques to the image may extend the application range of the rules. These rules can be summarized as a feature vector that distinguishes between foreground and background voxels. By training the feature vectors on foreground and background voxels, we obtained a classifier (Suykens and Vandewalle 1999; Cortes and Vapnik 1995) that was combined with our previous SparseTracer tool (Li et al. 2016) to give SparseTracer-Learned Feature Vector (ST-LFV). We have verified that ST-LFV accurately identifies weak signals from sparsely distributed neurites in light microscopic images and overcomes the identification difficulties caused by inhomogeneous backgrounds across different mouse brain regions. The observed rules used in ST-LFV can be applied to BigNeuron, DIADEM, and MOST datasets collected with various light microscopy techniques. In addition, the results demonstrated that ST-LFV significantly enhances the performance of SparseTracer in the large-scale tracing of sparsely distributed neurites.

## Methods

The components of the proposed ST-LFV are outlined in this section. First, we describe the method used to extract the feature vectors, which display the differences between the foreground and background voxels. Second, we introduce a support vector machine (SVM) to the feature vector space to construct a classifier that can detect weak signals (Suykens and Vandewalle 1999; Cortes and Vapnik 1995). Third, we integrate this constructed classifier into our previous SparseTracer (Li et al. 2016) for better neurite tracing performance. We also discuss the parameter selection procedure for ST-LFV and validate the proposed mechanism.

### Feature Extraction for Identifying Weak Signals

The extraction of representative image features is based on several assumptions about the images. Our assumptions are that the shape of a neurite can be described by a series of touching cylinders; that the background is locally smooth; and that, in a small local region, the foreground and background can be identified using a threshold value. For a given voxel, we extract its features using the image intensities of neighboring regions. This extraction includes three steps: i) set a series of threshold values for labeling the connected components of a given point; ii) generate the connected components using the threshold values; iii) use the generated components to construct the feature vector of this point. In the following, we describe how to extract features and explain why the extracted features are consistent with our assumptions.

**Step i)** Set a series of descending threshold values for labeling the neighboring regions of a point. For a given point p*, its corresponding threshold values are calculated by

1 $$ thr(m)=\Big\{{\displaystyle \begin{array}{c}\left(1-m{c}_1\right)s\left({p}^{\ast}\right)\kern0.5em if\ {c}_1s\left({p}^{\ast}\right)\ge {c}_2\\ {}\kern2.75em s\left({p}^{\ast}\right)-m{c}_2\kern0.5em otherwise\kern5.25em \end{array}} $$

where p* is 3D coordinates of point. For simplicity, we also denote the point by p*. When the coordinate elements of p* are integers, p* is regarded as a voxel. *s*(p*) is the weighted average image intensity of p* and its neighboring voxels; *c*_1_ and *c*_2_ are two predetermined constants, *c*_1_ = 0.025 and *c*_2_ = 1.5; *m* is an integer ranging from 0 to 8; and *thr*(*m*) is a threshold value that decreases as *m* increases. *s*(p*) is calculated by

2 $$ s\left({p}^{\ast}\right)=\frac{\sum_{p\widehat{I}T\left[{p}^{\ast}\right]}\mathit{\exp}\left(-\frac{1}{2}{\left\Vert p-{p}^{\ast}\right\Vert}_2^2\right)s(p)}{\sum_{p\widehat{I}T\left[{p}^{\ast}\right]}\mathit{\exp}\left(-\frac{1}{2}{\left\Vert p-{p}^{\ast}\right\Vert}_2^2\right)} $$

where *T* is the voxel set that includes the voxel [p*] and its 6-voxel neighborhood; [] represents the operation of rounding the coordinates of a point to its nearest values; *p* has the same definition as p*. *s*(*p*) is the intensity value of voxel *p*; $\left\Vert \right\Vert {}_2{}^2$ represents the 2-norm.

Note that the threshold value *thr*(*m*) is codetermined by 1-*mc*_1_ and the given voxel. To simplify the form of this expression, we regard the threshold value as a function of 1-*mc*_1_, where *m* = 0, 1, …, 8, and call 1-*mc*_1_ the invariable ratio. For small values of *s*(p*), *thr*(*m*) in the first term of Eq. (1) decreases slowly as *m* increases. To prevent this, we set a lower bound (*c*_2_ = 1.5) to overcome the decreased amplitude of the threshold values (second term in Eq. (1)).

**Fig. 2:**
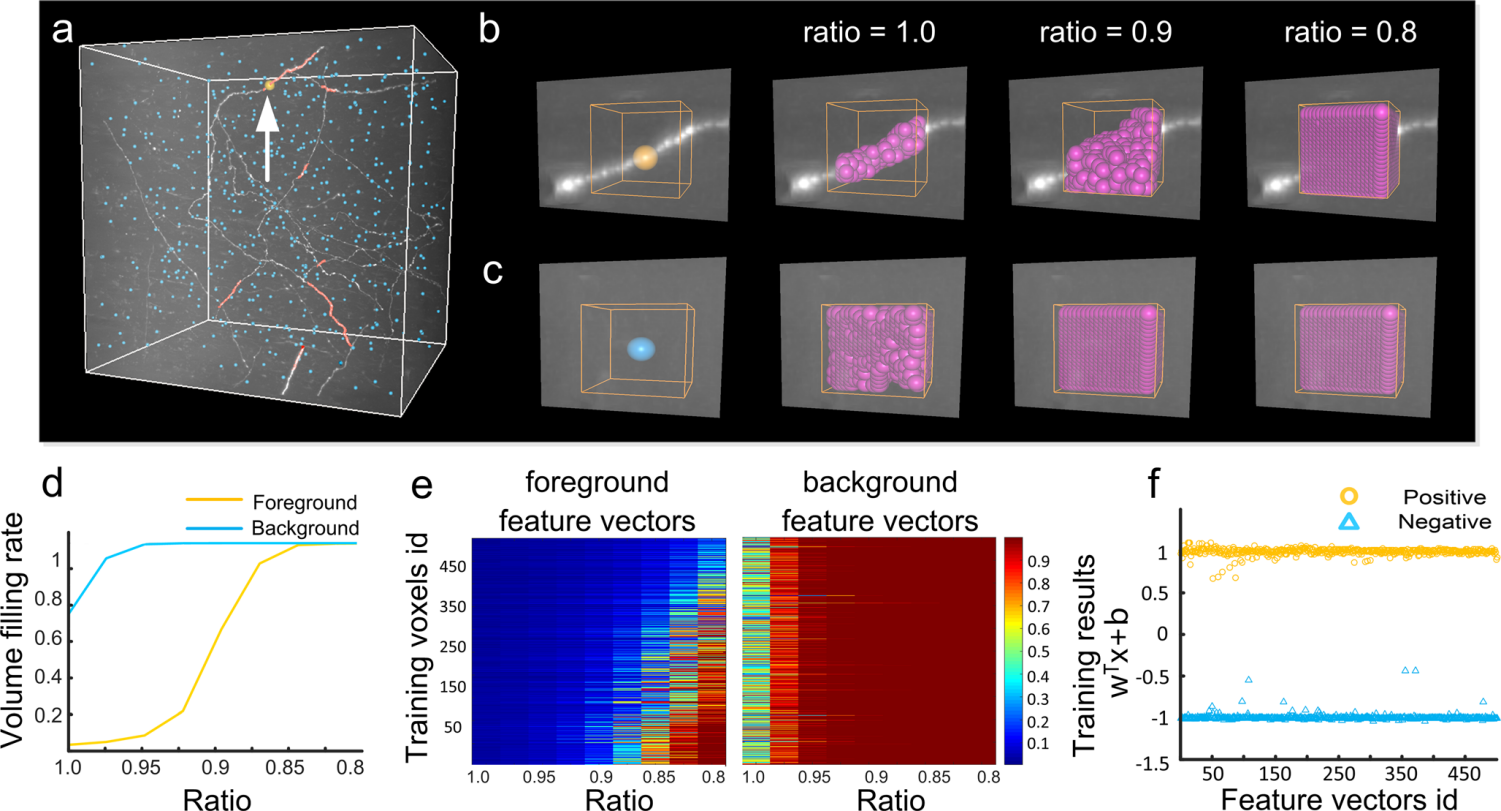
Illustration of feature vector extraction. **a** An image dataset including the labeled foreground voxels (light red) and background voxels (light blue). The foreground voxel (orange) is labeled with an arrow and its corresponding regions are shown in (b). **b** Calculating the regions of the selected foreground voxel (yellow) respective to different ratios. The neighborhood and the regions of this voxel are labeled by the yellow cubic and purple points, respectively. **c** Same as (b) for the selected background voxel (blue). **d** The foreground (yellow line) and background (blue line) feature vectors calculated from the given voxels in (b) and (c), respectively; **e** feature vectors of all labeled foreground (left) and background (right) voxels in (a); **f** generating an SVM classifier with feature vectors from (e). The positive (yellow circles) and negative (blue triangles) results correspond to the foreground and background feature vectors in (e), respectively

**Step ii)** Extract the connected components of a given point with the threshold values. For the given point p* and a threshold value *thr*(*m*), we use the region growing method to generate a connected component in which the voxels connect with each other and have image intensities greater than *thr*(*m*). The generated region is included in the pre-determined neighborhood *N*(p*) (19 × 19 × 19 voxels) of the given point p*. The steps for generating the connected component are described below.

(**ii-a**) Set the initial seed as the point p* and label it with an arrow in Fig. 2a, and search for its neighboring voxels according to

3 $$ {G}_1(m)=\left\{p\in {N}_1\subset N\left({p}^{\ast}\right)|s(p)> thr(m)\right\} $$

where *N*_1_ is the 26-voxel neighborhood of point p* with 3D coordinates *x*-, *y*-, and *z*-. The voxel [p*] and the searched voxels with image intensities greater than *thr*(*m*) are labeled. These labeled voxels form *G*_1_(*m*). [p*] rounds each element of p* to the nearest integer.

(**ii-b**) In the unlabeled region of *N*(p*), search for the 26-voxel neighborhoods of every voxel in the set *G*_*1*_(*m*), denoted by *N*_*2*_. According to *N*_2_ and the threshold *thr*(*m*), use Eq. (3) to generate *G*_*2*_(*m*) and then label the resulting set of voxels.

(**ii-c**) Repeat step **ii-b** until no new voxel sets can be generated in N(p*). The labeled sets *G*_1_(*m*), *G*_2_(*m*), … form the connected components of p* with respect to the threshold *thr(m)*, denoted by *G*(*thr*(*m*)).

Figures 2b, c illustrate how to obtain the connected regions of a foreground point and a background voxel under three threshold values, respectively. The size of the connected region of a given point depends on the threshold value *thr*(*m*), which is codetermined by the ratio 1-*mc*_1_ and the weighted average of the imaging intensities (Eqs. (1) & (2)). Thus, identical ratios, i.e., 1.0, 0.9, 0.8, do not indicate the same threshold values in the extraction of the connected regions.

**Step iii)** Calculate the feature vector of a given point. For the given point p*, we can obtain nine connected regions with respect to the threshold value *thr*(*m*), *m* = 0, 1, ..., 8. We define the volume filling rate as the ratio of the connected component volume (number of voxels) and the neighborhood region volume, given by

4 $$ {r}_m=\frac{\varOmega \left(G\left( thr(m)\right)\right)}{\varOmega \left(N\left({p}^{\ast}\right)\right)}\kern0.5em ,m=0,1,...,8 $$

where *Ω*(·) is the total number of voxels in a region and *r*_*m*_ represents the volume filling rate and is the *m*^*th*^ element of the feature vector ***x*** of the given point p*.

We explain why the extracted features of a point are consistent with our assumptions. If a point belongs to the background, the volume filling rate in the feature vector will rapidly increase to 1.0 because of the smoothness of the local background. For the feature vector of a foreground point, the volume filling rate will increase much more slowly, and may not even reach 1.0, as the cylindrical shape of a neurite takes up a small amount of space in its neighborhood and the intensities of the foreground and background voxels will be different. The differences between foreground and background feature vectors are illustrated in Fig. 2d.

### SVM Classifier Used to Identify Weak Signals

This section first describes the automatic extraction of training sets from neuronal images, and then explains how to build the SVM classifier after obtaining the training set.

In a supervised learning framework, a training set is necessary. Here, the training set contains the feature vectors of the foreground and background points. The automatic generation of training sets requires some foreground and background points to be obtained computationally, which may be practical for the following reasons. Existing tracing methods can identify weak signals at a certain level, which can provide foreground points. We used our SparseTracer tool (Li et al. 2016) to trace neurites and extract foreground points from the traced results. The traced results are composed of a series of points in which adjacent points are connected, providing the skeleton of a neurite. These skeleton points can be recognized as foreground points (Chen et al. 2015). If fewer than 500 skeleton points are selected, we calculate the feature vectors of all skeleton points. Otherwise, we acquired the signal intensities of these points in ascending order, and then chose those skeleton points having mid-level signal intensities. Finally, we calculated the feature vectors corresponding to the selected skeleton points. These feature vectors constitute the positive training set, denoted by *S*_*train*_ (left of Fig. 2e).

To obtain negative training samples, we randomly (from a uniform distribution) selected points from neuronal images that have the same number of positive training samples. We calculated their feature vectors (i.e. the negative training samples), denoted by *B*_*train*_ (right of Fig. 2e). The selected points may include a few foreground voxels, indicating that the negative training set contains some positive training samples. However, this selection is reasonable because, in most cases, the foreground voxels occupy less than 0.1% of the total number of voxels in neuronal images, according to our calculations (not shown). Thus, the number of foreground vector features included in the negative training set is negligible. In addition, SVM can tolerate a certain degree of error in constructing the training set. We identified whether a feature vector in the negative training set *B*_*train*_ is an outlier (the foreground feature vector) by measuring two degrees of similarity: one is the inner product between this feature vector and the mean values of *B*_*train*_, and the other is the inner product between this feature vector and the mean values of *S*_*train*_. If the former is larger than the latter, the vector is regarded as an outlier and is deleted from *B*_*train*_. The vectors remaining in the dataset comprise the negative training samples.

To simplify the description, we used {*y*_*k*_, ***x***_***k***_}, *k =* 1, 2*, …, K* to denote the positive and negative training sets. Here, *y*_*k*_ = 1 or − 1, ***x***_***k***_ is a feature vector, and *K* is equal to the number of training feature vectors in both *S*_*train*_ and *B*_*train*_. If *y*_*k*_ = 1, ***x***_***k***_ is positive and equal to an element in *S*_*train*_. Otherwise, ***x***_***k***_ is equal to an element in *B*_*train*_. After obtaining the training set, we introduced a linear SVM (Suykens and Vandewalle 1999; Cortes and Vapnik 1995) to build a supervised classifier. This classifier distinguishes between foreground and background voxels, and can be written as

5 $$ {\displaystyle \begin{array}{l}\underset{w,b,e}{\min }F\left(\mathbf{w},b,e\right)=\frac{1}{2}{\mathbf{w}}^T\mathbf{w}+\gamma \frac{1}{2}{\sum}_{k=1}^K{e}_k^2\\ {} subject\kern0.17em to\;{y}_k\left[{\mathbf{w}}^T{\mathbf{x}}_k+b\right]=1-{e}_k,k=1,2,...,K\kern1em \end{array}} $$

where ${\left\{{y}_k,{\mathbf{x}}_k\right\}}_{k=1}^K$ are the training samples described above, and ***x***_***k***_ refers to the *k*^*th*^ feature vector. Variable *e*_*k*_ represents the error term. γ is used to control the tradeoff between the training error and generalization ability. The optimization problem in Eq. (5) is then converted into an unconstrained optimization problem (Rockafellar 1973; Hestenes 1969):

6 $$ \underset{w,b,e;\alpha }{\min }L\left(\mathbf{w},b,e;\alpha \right)=F\left(\mathbf{w},b,e\right)-{\sum}_{k=1}^K{\alpha}_k\left\{{y}_k\left[{\mathbf{w}}^T{\mathbf{x}}_k+b\right]-1+{e}_k\right\} $$

where α_k_ is the Lagrange multiplier. Using the Kuhn–Tucker conditions, we could obtain the optimal solution (Suykens and Vandewalle 1999), and the corresponding supervised classifier can be denoted by

7 $$ R\left(\mathbf{x}\right)=\mathit{\operatorname{sgn}}\left({\sum}_{k=1}^K{\alpha_k}^{\ast }{y}_k{\mathbf{x}}_k^T\mathbf{x}+{b}^{\ast}\right)=\mathit{\operatorname{sgn}}\left({\sum}_{k=1}^K{\mathbf{w}}^{\ast T}\mathbf{x}+{b}^{\ast}\right) $$

where **x** represents the input feature vector, α_k_^*^and the coefficients w*, b* are obtained by solving the optimization problem (6). *sgn*() represents the sign function. If w*^T^**x** + b* > 0, the input belongs to the positively labeled set; otherwise, it belongs to the negatively labeled set. Applying the classifier to the training samples shows that most of the positive and negative values are close to 1 and − 1, respectively (Fig. 2f), which illustrates the large differences in the feature vectors of the foreground and background points (Fig. 2e).

### Using the Identification Model for Neurite Tracing

**Fig. 3:**
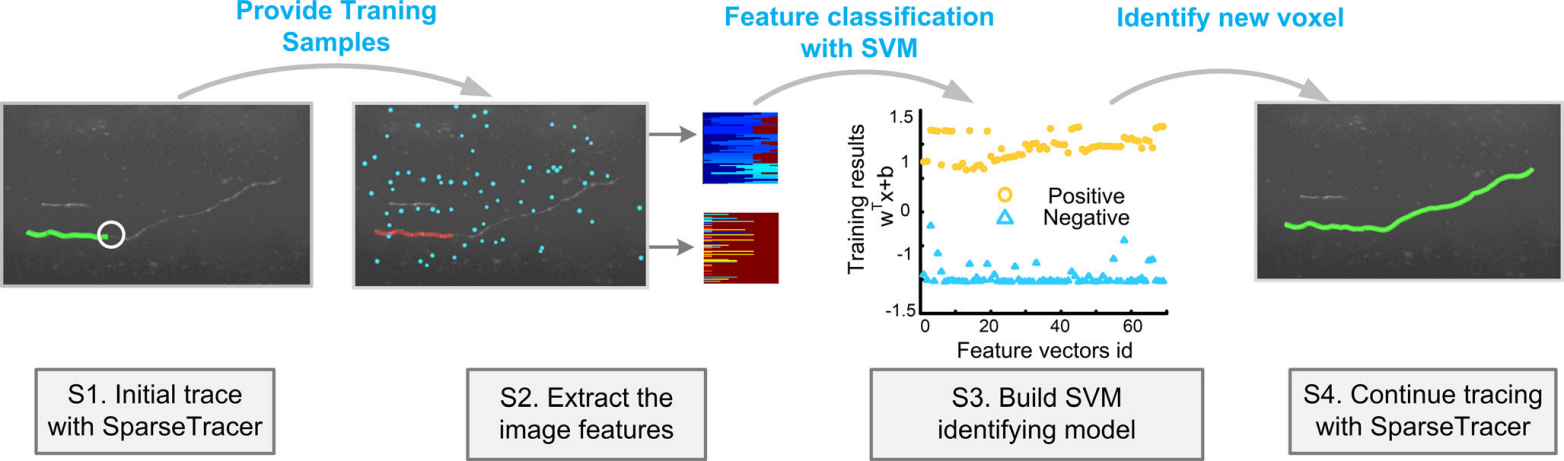
Pipeline of neurite tracing with ST-LFV. Step 1: Acquire the initial skeleton (green curve) of the neurite using SparseTracer. The site where SparseTracer fails is labeled with a circle; Step 2: Extract the feature vectors from the labeled voxels. These labeled voxels include foreground voxels (red) on the initial skeleton in Step 1 and background voxels (blue), and their corresponding feature vectors are located in the upper and lower panels, respectively; Step 3: Construct a SVM classifier with feature vectors in S2; Step 4: Use the classifier to identify weak signals and continue tracing. The final reconstruction (green) is thus obtained

Neurite tracing is the process of obtaining the skeleton of a neurite. A key component of neurite tracing is the accurate identification of foreground points. When tracing a neurite, if the current tracing point is identified as a background point, the tracing will be terminated. We applied the identification model described above to our SparseTracer tool to obtain better neurite tracing results. The pipeline is described as follows (Fig. 3)*.*

**Step 1)** Use SparseTracer to trace the neurite. When the point *p*_*n + 1*_ is identified as a background point, tracing stops and an initial skeleton is generated, represented by*P* = {*p*_1_, *p*_2_, ..., *p*_*i*_, ..., *p*_*n*_}, where *p*_*i*_ is the *i*^th^ point on the skeleton.

**Step 2)** Extract the feature vectors of foreground and background points separately. These form the positive and negative training sets, respectively. Note that the foreground points are the skeleton points of traced neurites generated with SparseTracer.

**Step 3)** Obtain the SVM classifier with the training set.

**Step 4)** Apply the obtained classifier to the identification of points *p*_*n*_ and *p*_*n + 1*_. If one of these two points is identified as a foreground point, continue tracing with SparseTracer, and go to **step 5**). Otherwise, go to **Step 6)**.

**Step 5)** In the tracing process, if the last two tracing points are identified as a background point with SparseTracer, the SVM classifier will automatically activate. If both these two points are identified as the background points, terminate this tracing and go to **Step 6**); otherwise, continue tracing with SparseTracer and SVM classifier.

**Step 6**) If point *p*_*1*_ is not a branching point, carry out the same tracing and identifying procedure in **Step 4**) and **Step 5**) for the points *p*_2_ and *p*_1_, until the termination condition is satisfied, namely, the last two tracing points are identified as the background points with the SVM classifier. Otherwise, finish this neurite tracing.

The above steps describe how our identification model and SparseTracer collaborate to achieve better neurite tracing. Note that our identification model learns from the characteristics of an image stack, and is only applied in this image stack. If the neurites in a given image stack cannot be traced completely after one iteration, we design a strategy in which, once our constructed classifier detects some foreground points with weak signal intensities, the positive training set can be updated and used to build a new classifier in the next tracing iteration. This strategy helps to provide nearly complete neurite reconstructions for a given image stack.

### Parameter Settings in the Identification Model

To construct the identification model for detecting weak signals, certain key parameters must be pre-determined, including the size of the training set, the ratios used in feature vector extraction, and the size of the neighborhood.

#### Size of the Training Set

The positive training set depends on the tracing results. If the total number of foreground points in the traced neurites is less than a pre-determined threshold (500 in this study), we selected all foreground points and calculated their feature vectors to form the positive training set. In this case, though the size of the positive training set is small (dozens of points), the SVM classifier still behaves well. This is why we do not use upsampling to increase the size of the training set. When the traced neurites include many points, numerous positive feature vectors can be generated. In this case, the upper limit for the number of feature vectors in the positive training set is 500. This threshold is based on a tradeoff between computational cost and classification performance, as the inclusion of more training samples may not improve identification performance. This selection ensures that the time required to identify weak signals is approximately the same as that required for neurite tracing. In addition, balanced training sets are ideal for supervised SVM classifiers, and so the negative training set is of similar size as the positive training set (Tang et al. 2009).

#### Ratios Used in Feature Vector Extraction

The feature vector of a point depends on certain ratio settings, as described in Eq. (1). In our analysis, the ratios range from 0.8–1, and the difference between two adjacent ratios is *c*_1_ *=* 0.025. The choice of a small *c*_1_ is based on one of our assumptions, namely, the local background is smooth. Consequently, a slight decrease in the ratio (i.e., a small *c*_*1*_) can fill the entire neighborhood of a background point through region growing, and the corresponding elements of its feature vector will be equal to 1. Therefore, a small value of *c*_1_ can capture the smoothness of the background. A rapidly decreasing ratio (i.e., a large *c*_1_) would indicate lower threshold values. With these lower thresholds, the region of a weak signal point would quickly fill its entire neighborhood, and thus the features of the neurite’s morphology would not be captured. The parameter *c*_2_ covers situations in which the background intensity is very low and *c*_1_ is not sufficient to achieve an appropriate granularity in ratios for feature vector extraction. Similar to *c*_1_, the selection of *c*_2_ aims to maintain the ability to detect weak neurite signals while identifying local background smoothness. Overall, the selection of these two parameters is intended to capture the feature differences between weak signal voxels and background regions.

#### Size of the Neighborhood in Feature Vector Extraction

In feature vector extraction, the neighborhood of a point contains 19 × 19 × 19 voxels. This is based on the following considerations: if the size of a neighborhood is small, the local morphology of a neurite extracted with a relatively low threshold may, in some situations, span the entire neighborhood, preventing the capture of its local morphology. However, a large neighborhood gives rise to the need for highly complex computations to obtain the region, which is a key step in feature vector extraction. Considering the diameter of a neurite (less than 5 μm) (De Paola et al. 2006; Stettler et al. 2006; Loopuijt et al. 2007) and the voxel size (0.5 × 0.5 × 0.5 μm^3^~ 2 × 2 × 2 μm^3^), we set the neighborhood range to be 19 × 19 × 19 voxels. This setting satisfies the condition that the local morphology of a neurite occupies a small portion of the neighborhood. All of the parameters discussed in this subsection remain unchanged throughout our analysis.

### Multi-Fold Cross-Validation of SVM Classifier

To validate the effectiveness of the constructed SVM classifier in our identification model, we used multi-fold cross-validation (Kohavi 1995). The procedure of cross-validation is as follows: in the image stack, we used our method to generate a data set containing 500 foreground feature vectors and 500 background feature vectors. The data set was randomly partitioned into 10 equally sized subsets in which both the foreground and positive feature vectors have the same number. Of the 10 subsets, a single subset was retained as testing data and the remaining 9 subsets were used for training data. This process was then repeated 10 times, and correspondingly, 10 testing errors were generated. We averaged these testing errors to evaluate the SVM classifier (**See Table****S1**).

### Evaluation of Automated Neurite Tracing Methods

The precision and recall rates are often used to quantify the difference between the automatic and manual reconstruction given by a series of traced skeleton points. Here, each skeleton point has three-dimensional coordinates, and also can be regarded as a voxel if its coordinate elements are integers. These evaluation measurements were used in our previous studies (Quan et al. 2016; Li et al. 2016). The precision and recall are computed according to the numbers of true positive points. A true positive point is defined as follows: For any given point on the automatic reconstruction, find its nearest point on the manual reconstruction. If the distance between the given point and the found point is less than a pre-determined threshold (6 μm in this study), the given point is considered to be a true positive point. The pre-determined distance threshold judges whether a point in one skeleton is equated to a point in another skeleton or not. The parameter is set based on the consideration of the morphological characteristics of thick dendrites and total length of a neuron. According to our previous work (Quan et al. 2016), the evaluation results change slightly when this parameter ranges from 6 μm to 10 μm. The precision is then defined as the ratio of the number of true positive points to the total number of points in the automated reconstruction. The recall is defined as the ratio of the number of true positive points to the total number of points in the manual reconstruction.

## Results

To evaluate the performance of the proposed ST-LFV, the fMOST (Gong et al. 2013), DIADEM (Brown et al. 2011), and BigNeuron (Peng et al. 2015) datasets were used. The fMOST dataset includes typical sub-blocks from different mouse brain regions collected with the fMOST imaging system (Gong et al. 2013) using a voxel size of 0.3 × 0.3 × 1 μm^3^. These voxels were automatically merged with sizes in the range from 0.5 × 0.5 × 0.5 μm^3^ to 2 × 2 × 2 μm^3^. This range is suitable for our tool GTree using which our analysis was performed here. The DIADEM (www.diademchallenge.org) and BigNeuron (http://alleninstitute.org/bigneuron/data/) datasets are freely available; information about these datasets can be found on the respective websites. We performed experiments on a computer workstation (Intel® Xeon® CPU 3.46 GHz computing platform, Quadro K4000 3G GPU, 192 GB RAM, Windows 7). Our analysis involved two algorithms: an automatic tracing algorithm, SparseTracer, and the combination of SparseTracer and the learned feature vectors in ST-LFV. The proposed algorithms (SparseTracer and ST-LFV) are integrated into our software GTree (https://github.com/GTreeSoftware/GTree/releases). GTree is implemented using widely used open source standards and programming practices (C++ with ITK and VXL libraries, graphical user interfaces written with QT version 5.5.1 and VTK version 8.0) and uses a GNU GENERAL PUBLIC LICENSE (https://github.com/GTreeSoftware/GTree/blob/master/License.md). When we used SparseTracer to analyze each image stack, we carefully selected the parameters to ensure the optimal tracing results. When using ST-LFV, the default settings for the tracing parameters were used, as they can provide an initial training set in most situations; the identification parameters are fixed and discussed in detail in the Methods section.

**Fig. 4:**
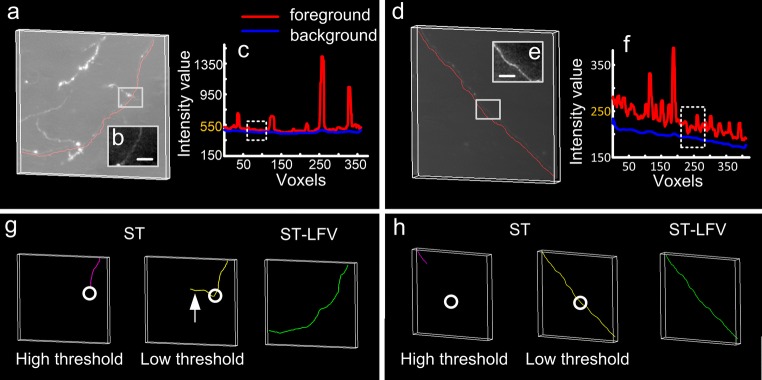
Performance on datasets with inhomogeneous backgrounds drawn from SparseTracer (ST) and ST-LFV. **a** One dataset with a manually traced neurite (red). Part of this traced neurite is labeled with a square. **b** Maximum projections of labeled regions in (a) through a depth of 10 μm, with a scale bar of 10 μm; **c** foreground (red) and background (blue) intensities of the traced neurite in (a). The intensities of the neurite in (b) are close to its background, labeled with a dashed square. **d** One dataset with a weak background and one similar to (a); **e** and **f** have similar descriptions as (b) and (c), respectively; **g** tracing results drawn from ST with a high threshold (purple) and a low threshold (yellow), and drawn from ST-LFV (green). The location of the weak neurite in (b) is labeled with circles. Over-traced results (arrow) drawn from ST with a low threshold; **h** similar to (g). Here, ‘high threshold’ refers to the default threshold set in the algorithm, which can provide robust tracing results in most cases; ‘low threshold’ refers to a well-chosen threshold that produces better tracing results in a specific case

We first demonstrated the ability of ST-LFV to trace neurites in inhomogeneous neuronal images. Two image stacks were selected from a whole brain imaging dataset and the relevant image characteristics were identified (Fig. 4a-f**)**. We evaluated the image quality of selected sub-blocks (Fig. 4b, e) using the contrast-to-noise ratio (CNR) (Song et al. 2004). The CNRs of the target areas (**Fig.****S1**) range from 1.53–2.68, indicating that some regions have small differences between their foreground and background intensities. However, by modifying the signal range in the visualization mode, we were able to discriminate the foreground from the background, and thus manual tracing could achieve the ground-truth results (red curves in Fig. 4a, d). We further calculated the image intensities of the skeleton points on manually traced neurites (red curves in Fig. 4a, d) from the original image and its estimated background image (Quan et al. 2013). In most cases, the background intensities (blue curve in Fig. 4c) estimated from Fig. 4a are 2–3 times larger than the foreground intensities (red curve in Fig. 4f) calculated from Fig. 4d. This indicates that the background intensities vary sharply in brain-wide imaging datasets. In this case, we compared the tracing results drawn using SparseTracer with those from ST-LFV (Fig. 4g, h). SparseTracer can trace the neurite well (Fig. 4d, h) with a suitable threshold. However, the same threshold is not applicable in other cases (Fig. 4a, g). In contrast, ST-LFV can attain trace results that are almost equal to the ground truth. We concluded that ST-LFV can overcome the influence of the inhomogeneous background to trace neurites successfully.

**Fig. 5:**
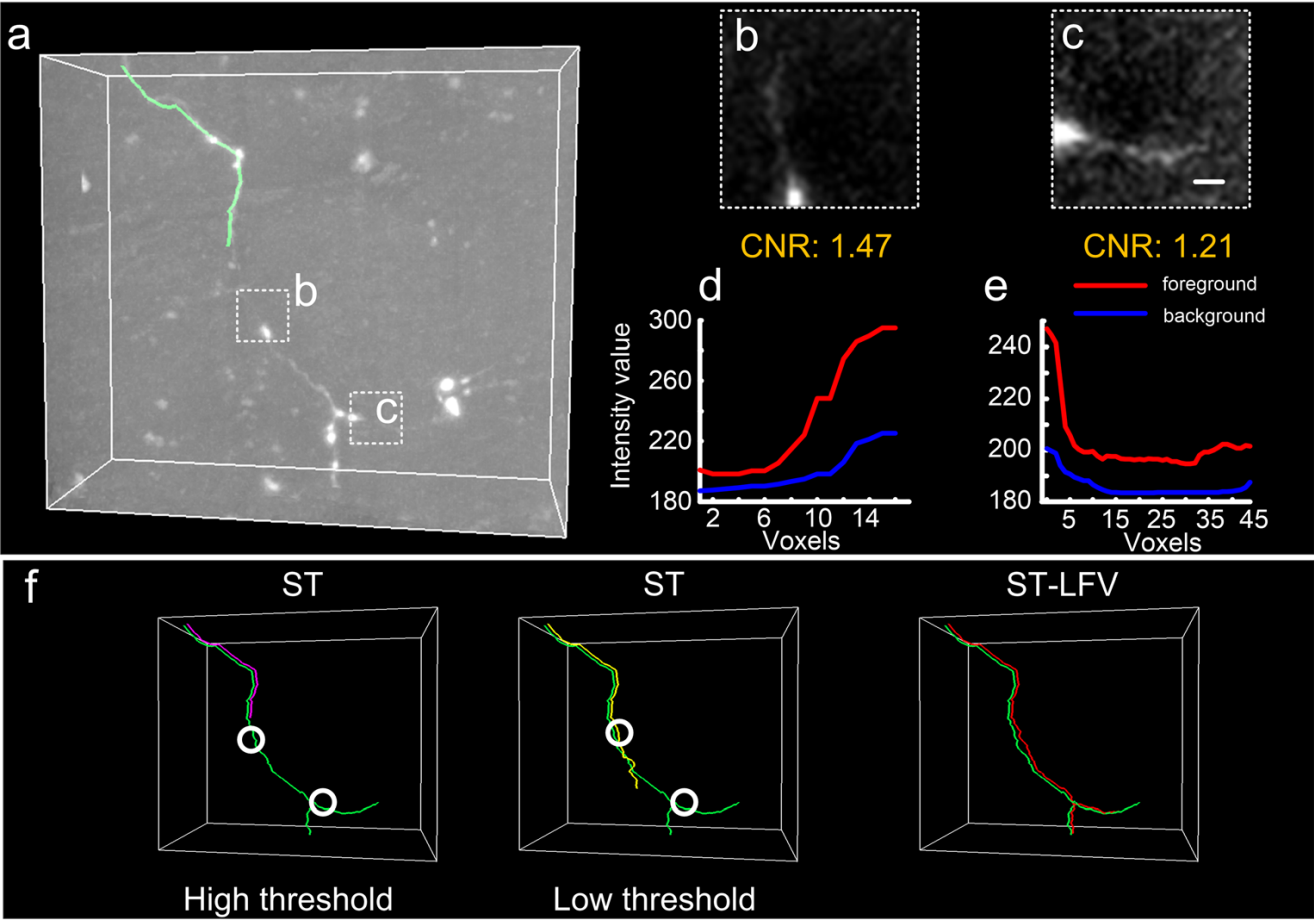
Performance on a dataset with weak neurites drawn from SparseTracer (ST) and ST-LFV. **a** Neurite and its initial tracing skeleton (green) drawn from ST. Two typical weak signal regions (dashed squares b and c) contain portions of this neurite; **b** and **c** are enlarged views of the labeled regions in (a). Both include maximum intensity projections through a depth of 10 μm, with a scale bar of 5 μm. **d** and **e** Foreground and background intensities of the neurites in (b) and (c), respectively; **f** tracing results drawn from ST with a high threshold (purple) and a low threshold (yellow), and from ST-LFV (red). The manual tracing results are labeled with green curves. The locations of the weak neurite in (b) and (c) are labeled with circles

We used an experimental dataset to verify the ability of ST-LFV to identify weak signals. The experimental dataset included a neurite with weak signals at several sites (Fig. 5a). We selected two sites and extracted their corresponding sub-blocks (Fig. 5b, c). These two sub-blocks had CNRs of 1.47 and 1.21, respectively. We manually traced the neurites in these two sub-blocks and computed the foreground and background values of the skeleton points in the traced neurites (Fig. 5d, e). These results (Fig. 5b-e) show that the neurites have weak signal intensities at some sites. SparseTracer cannot handle this case, tracing only parts of the neurite (left and middle panels in Fig. 5f), despite repeated efforts to select suitable thresholds. Compared with SparseTracer, ST-LFV yields good tracing results (red curve, right panel in Fig. 5f) that are broadly equivalent to the ground truth (green curve in Fig. 5f). ‘High threshold’ and ‘low threshold’ in Fig. 5f have the same meaning as in Figs. 4g, h, and are explained in the legend of Fig. 4.

**Fig. 6:**
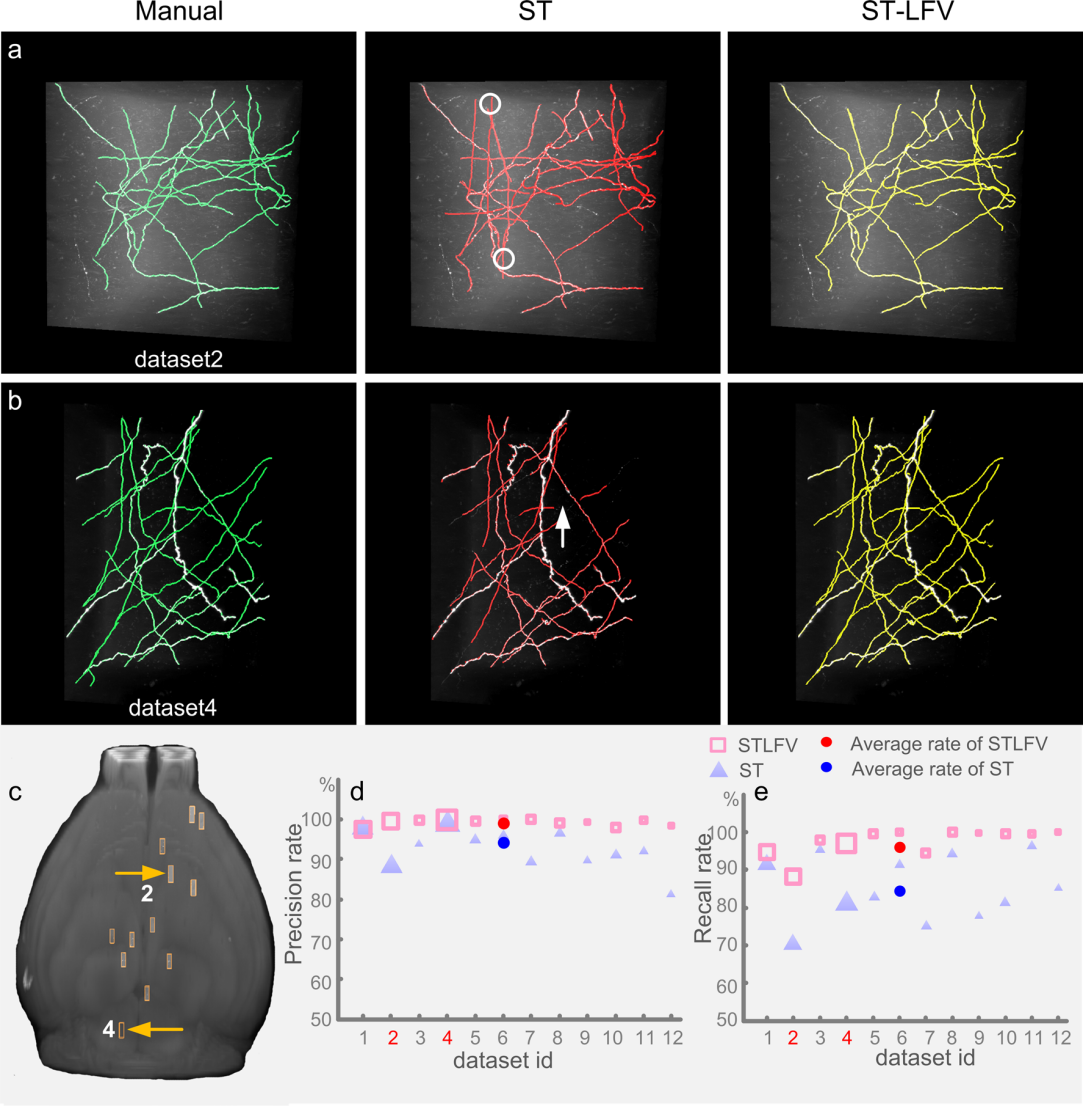
Comparisons of neurite tracing results drawn from SparseTracer (ST) and ST-LFV. **a** One typical dataset with its corresponding tracing results drawn manually (green), drawn with ST (red), and drawn with ST-LFV (yellow). Over-tracing results from ST are labeled with circles. **b** Another dataset with a weak background in which ST fails to trace part of a neurite (labeled with an arrow); **c** distribution of the dataset locations in the mouse brain. The locations of datasets (a) and (b) are labeled with yellow arrows; **d** and **e** are automatic tracing results from the datasets in (c) and are measured with a precision rate (d) and a recall rate (e), respectively. The red numbers 2 and 4 on the lateral axis represent the datasets shown in (a) and (b)

Furthermore, we showed that ST-LFV is superior to SparseTracer for tracing neurites using 12 experimental image stacks containing sub-blocks of 600 × 600 × 600 voxels. These datasets are clearly distributed among different brain regions in which the background intensities change obviously (**Fig.****S2**). Two typical datasets and their corresponding tracing results are presented in Fig. 6a, b. The sites of these two datasets are labeled with yellow arrows in Fig. 6c. The sites of the other ten datasets are also shown in Fig. 6c. When using SparseTracer to analyze these datasets, we selected the tracing threshold value that maximized the average tracing accuracy and used this setting for all datasets. This sometimes caused SparseTracer to produce more tracing results than manual reconstruction (white circles in middle panel, Fig. 6a). Unlike SparseTracer, ST-LFV generates the corresponding identification models for each dataset to identify untraced foreground points. We used multi-fold cross-validation (see section 2.5) to validate the constructed SVM classifier in the identification model (**Table****S1**). The highest error rate was 2.1% (dataset 2); the other datasets had error rates of less than 0.5%. The cross-validation results indicate the robustness of the SVM classifiers to image stacks from different brain regions. Furthermore, we quantified the tracing results from SparseTracer and ST-LFV (Fig. 6d, e). The average precision and recall rates were 93% and 86% for SparseTracer and 99% and 97% for ST-LFV, respectively. These results indicate that ST-LFV can achieve almost complete neurite reconstruction using our identification model.

**Fig. 7:**
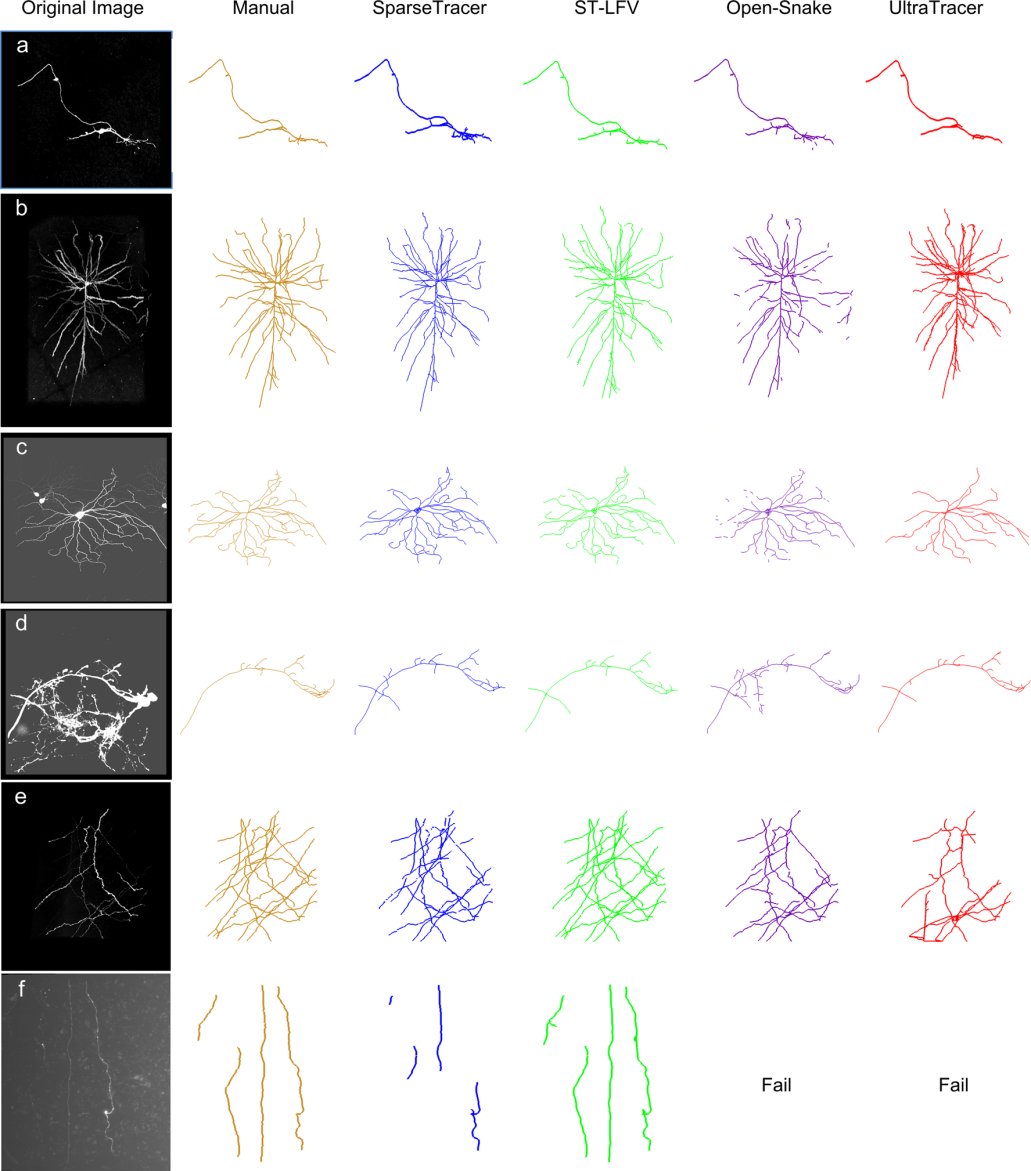
Comparisons of the reconstruction derived from manual, SparseTracer, ST-LFV, Open-Snake and UltraTracer. The first column of panels (**a**)-(**f**) show the original image stacks provided by DIADEM Challenge (dataset d, voxel size: 1 × 1 × 1 μm^3^), BigNeuron project (dataset b, voxel size: 0.18 × 0.18 × 0.5 μm^3^; datasets a and c, voxel size: 1 × 1 × 1 μm^3^) and MOST datasets (datasets e and f, voxel size: 0.3 × 0.3 × 1 μm^3^), respectively. The remaining columns of the panels are the reconstructions generated by manual, SparseTracer, ST-LFV, Open-Snake and UltraTracer, respectively

We also compared the reconstruction performances derived by using our previous method SparseTracer, our method ST-LFV, Open-Snake (Wang et al. 2011) and UltraTracer (Peng et al. 2017) on various datasets. The datasets are from BigNeuron project, DIADEM challenge and MOST data. According to the reconstruction and quantified results (Fig. 7**& Table****S2**), all the tested methods behaved well on datasets with simple neuron structure and clean background (Fig. 7a, b). Open-snake and UltraTracer showed their own advantages on some specific neurite structures (Fig. 7c, d), such as, neurons consisting of short, thick neurite segments (Fig. 7d). With regard to tracing axonal neurites with weak image intensity, SparseTracer, Open-snake and UltraTracer were challenged (Fig. 7e) and some of them even failed to cope with this case (Fig. 7f). Note that in the use of SpareseTracer, the tracing seeds were manually provided except for Fig. 7e. This is because the image (Fig. 7e) includes many separate neurites and manually selecting tracing seeds is relatively time consuming. These results provide some evidences that identifying the weak foreground in neuronal images is still a challenging problem for the state-of-the-art method. In this demonstration, ST-LFV can identify almost all axonal neurites.

**Fig. 8:**
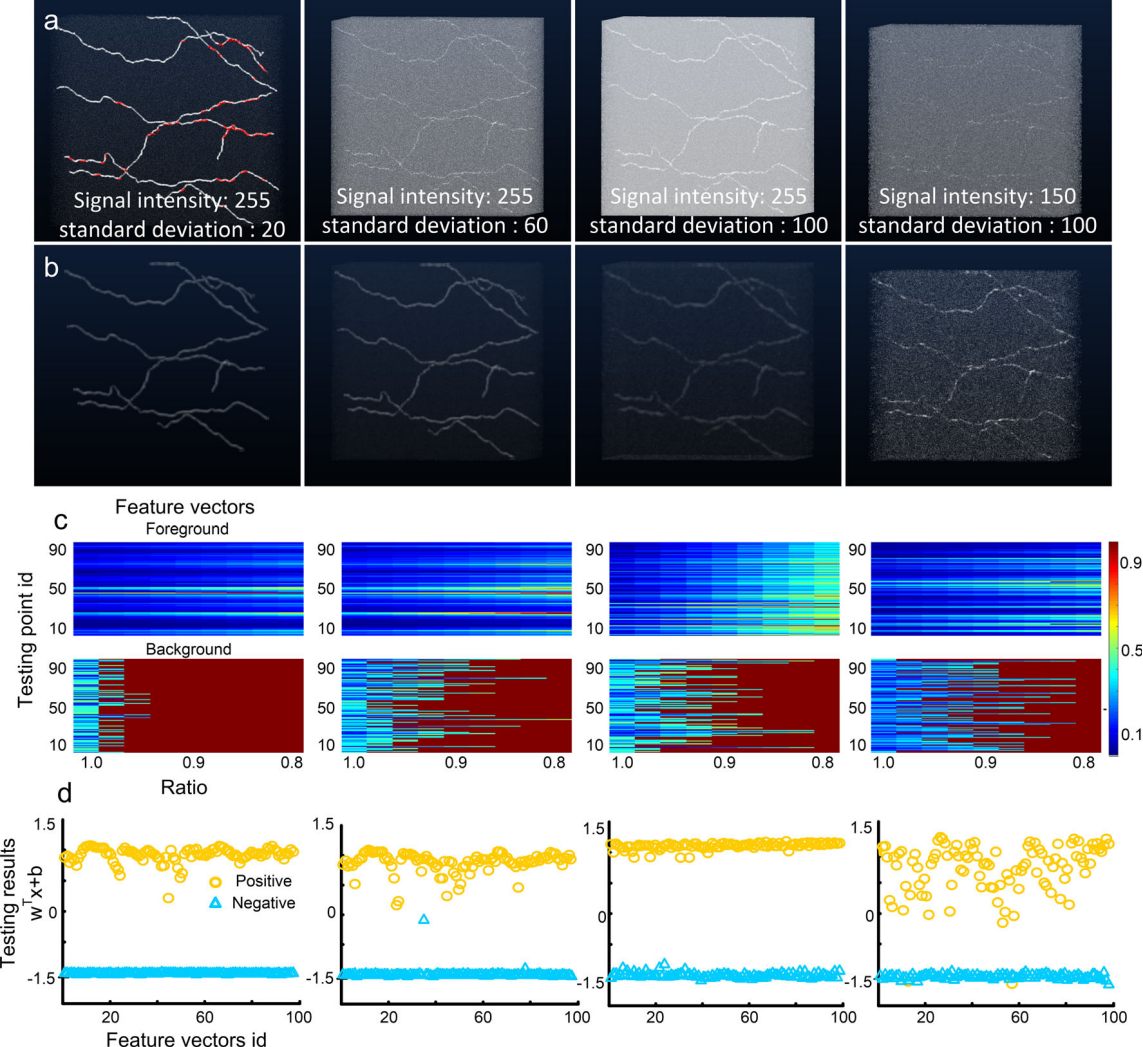
Feature vector extraction from filtered synthetic datasets. **a** Four image stacks with Gaussian noise. The red curves in (a) depict the initial tracing results. **b** Images in (a) filtered with an anisotropic denoising method; **c** foreground (upper panel) and background (bottom panel) feature vectors form the testing set and extracted from their corresponding filtered image stacks in (b); **d** differentiating the positive (yellow circles) and negative (blue triangles) feature vectors in the testing sets with the SVM classifiers. The classifiers are drawn from their corresponding image stacks

Next, we demonstrated that our identification model is not limited to images without a smooth background. Synthetic datasets were used for this purpose. Each dataset consisted of 517 × 515 × 517 voxels and contained several neurites. The signal intensity of the first three images in Fig. 8a is 255, but with different noise levels (Gaussian white noise with zero mean and standard deviation of 20, 60, or 100). The last image corresponds to signal and noise intensities of 150 and 100, respectively. These four images have CNRs (Welvaert and Rosseel 2013) of 12.75, 4.25, 2.55, and 1.5, respectively. An anisotropic Rudin-Osher-Fatemi (ROF) denoising method (Goldstein and Osher 2009; Rudin et al. 1992) was applied to smooth the image stack (Fig. 8b). We then validated the identification model drawn from the corresponding smoothed image stacks. When building the model, the positive training sets were calculated from the initial traced results (red curves in left-top panel, Fig. 8a). The negative training sets were formed of randomly chosen voxels. We manually checked the availability of the testing sets, i.e., that the positive and negative feature vectors corresponded to foreground and background voxels, respectively. The voxels from these four smoothed image stacks (Fig. 8b) had the same coordinates, and each smoothed image stack generated a testing set (Fig. 8c). In the testing sets, even with the high noise level, the positive and negative feature vectors were still separable (right panel in Fig. 8c). The results indicate that our feature vectors differentiate the foreground and background sufficiently well to handle images with nonsmooth backgrounds using an appropriate denoising method. We evaluated the error rate of the SVM classifiers on four testing sets (Fig. 8d). The highest error rate is 3% for the data with the highest noise level (fourth image in Fig. 8b). From these results, we could conclude that our identification model can be applied to image stacks with a nonsmooth background.

**Fig. 9:**
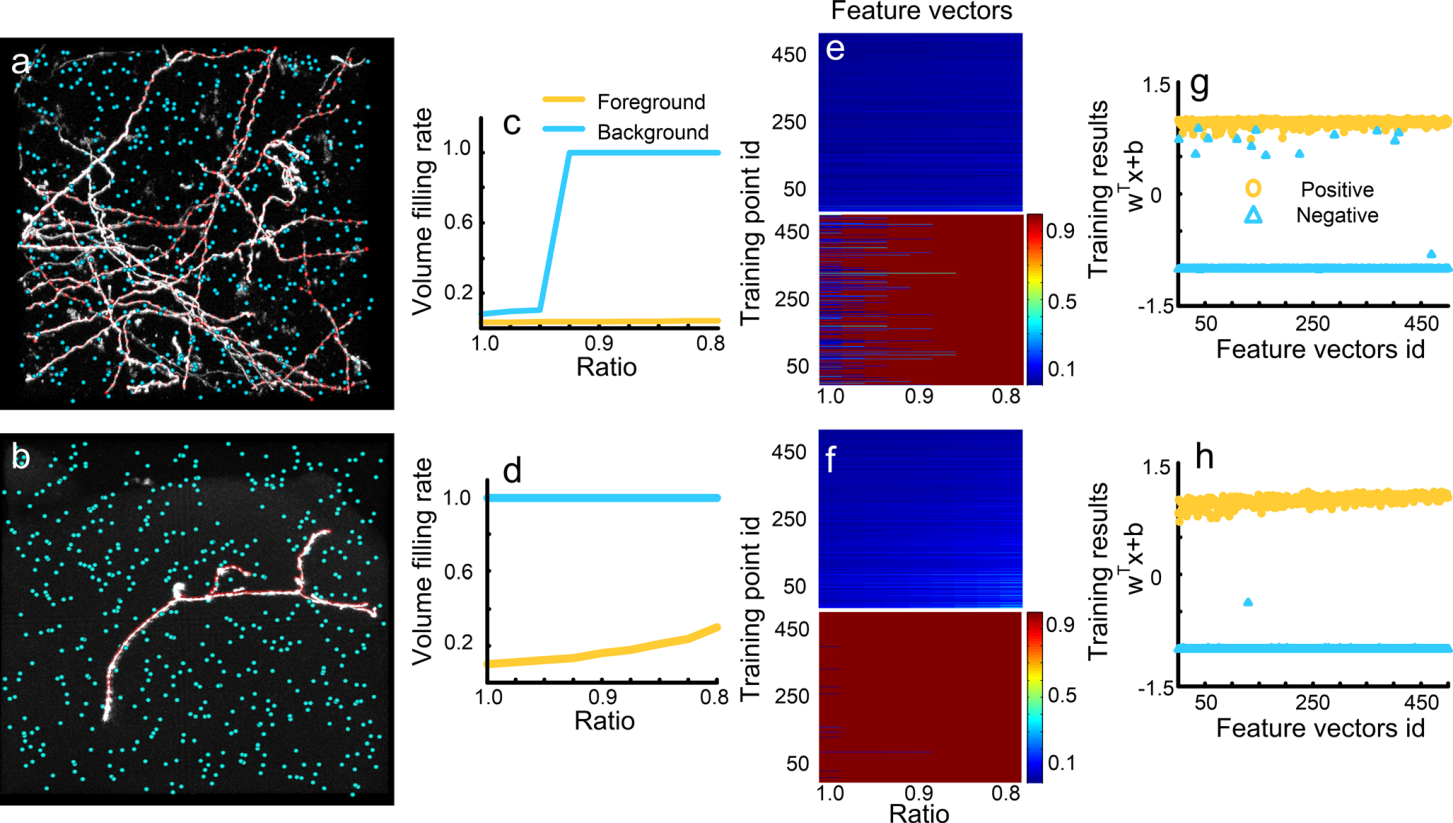
Feature vector extraction from DIADEM datasets. **a** and **b** are two datasets, one from the Neocortical Layer 6 Axons dataset and the other from the Olfactory Projection Fibers dataset. The foreground (red) and background (blue) voxels are used for feature extraction. **c** and **d** Foreground (yellow) and background (blue) feature vectors calculated from two labeled voxels in (a) and (b), respectively; **e** and **f** are feature vectors of all labeled foreground (upper) and background (bottom) voxels in (a) and (b), respectively; **g** and **h** show the SVM classifiers generated with feature vectors from (e) and (f), respectively. The positive (yellow circles) and negative (blue triangles) results correspond to foreground and background feature vectors, respectively

We investigated whether our assumptions were applicable to DIADEM datasets. Two datasets were used for this purpose. The dataset shown in Fig. 9a (Neocortical Layer 6 Axons dataset) was imaged by a two-photon microscope. The other in Fig. 9b (OP dataset) was imaged by 2-channel confocal microscopy. We extracted the foreground points and background points and calculated the corresponding feature vectors. The calculated feature vectors (Fig. 9c-f) illustrate the large feature differences between the extracted foreground and extracted background points. These differences result in a classifier for detecting weak signals with a low training error (1.3% for Fig. 9g and 0% for Fig. 9h, respectively). Note that, in generating the training set, the extracted background points included a few points located in regions adjacent to or at the boundaries of neurites (Fig. 9a). This may reduce the power of the classifier. We quantified this negative influence by applying cross-validation and the corresponding error rate is 0.75%. For the clean image background in Fig. 9b, the corresponding error rate is 0.05%.

**Fig. 10:**
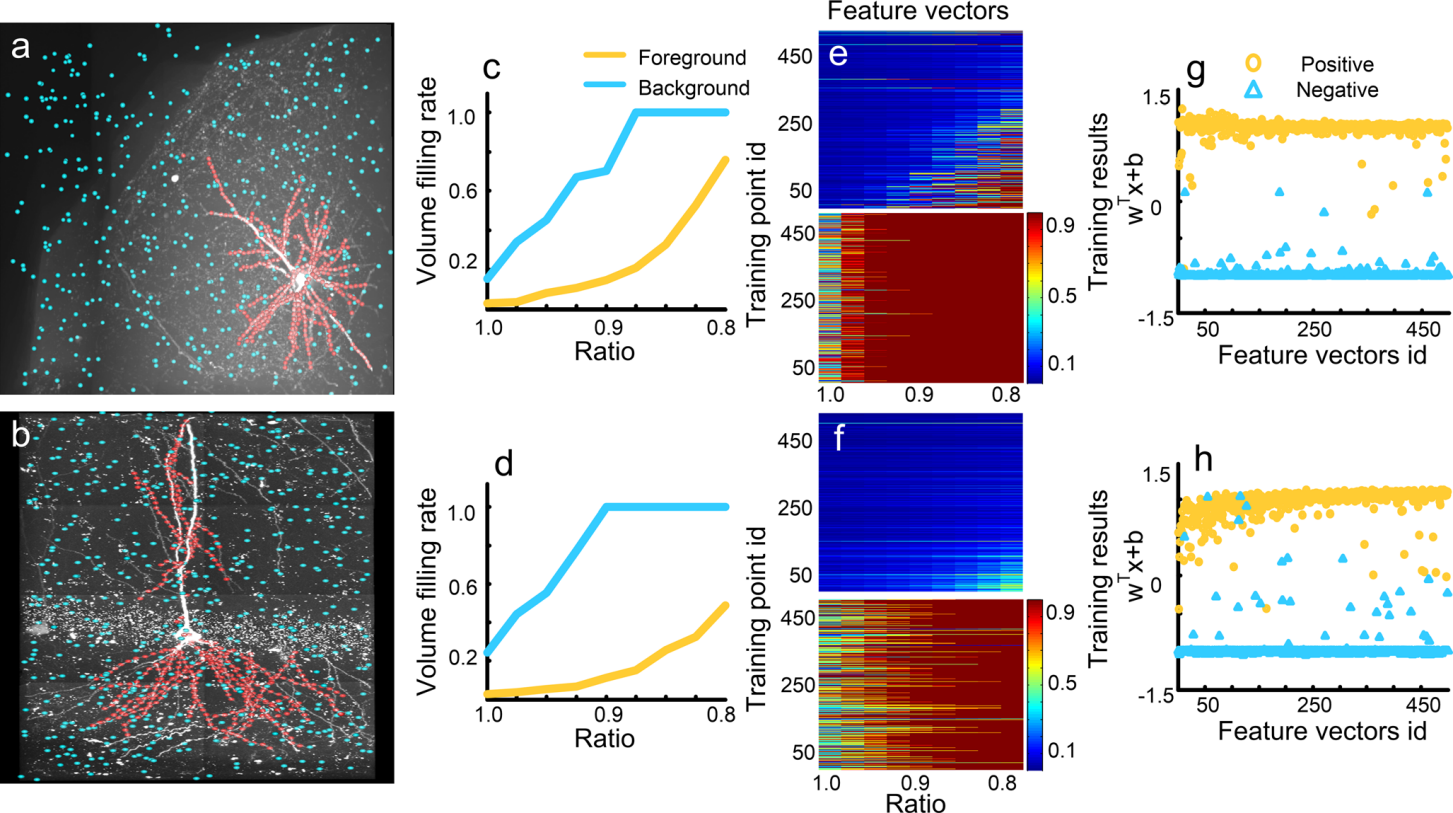
Feature vector extraction from BigNeuron datasets. **a** and **b** are two datasets, one from the checked6_mouse_tufts dataset and the other from the checked_mouse_korea dataset. The foreground (red) and background (blue) voxels are used for feature extraction. **c** and **d** Foreground (yellow) and background (blue) feature vectors calculated from two labeled voxels in (a) and (b), respectively; **e** and **f** are feature vectors of all labeled foreground (upper) and background (bottom) voxels in (a) and (b), respectively; **g** and **h** SVM classifiers generated with feature vectors from (e) and (f), respectively

We also demonstrated that our assumptions hold for BigNeuron data using two typical datasets (checked6_mouse_tufts and checked_mouse_korea) with a noisy background (Fig. 10a, b). Despite the presence of such noise, the foreground and background feature vectors are different and can be easily classified into two groups (Fig. 10c-f). These feature vectors were used to derive classifiers with training error rates of 0.4% (Fig. 10g) and 1.2% (Fig. 10h). The training errors can be attributed to the interference of the noisy background. We further validated the identification model using cross-validation. The estimated error rates are 0.05% and 0.1%, respectively. The low error rates indicate large differences between the features of the foreground and background points. These results indicate that our assumptions are consistent with the features of the BigNeuron datasets, and that the generated model is valid.

**Fig. 11:**
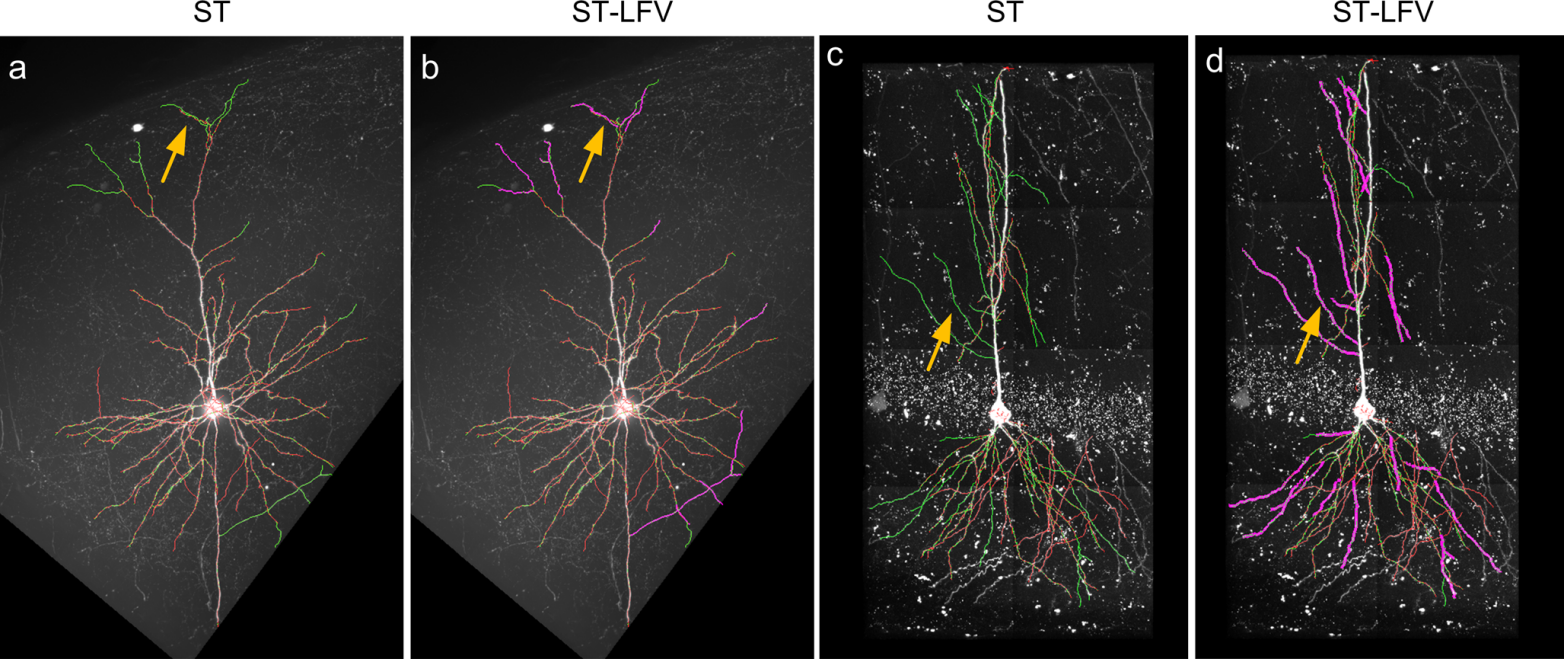
Tracing performance on two BigNeuron datasets drawn using SparseTracer (ST) and ST-LFV. **a** Tracing results drawn using ST (red). Some neurites cannot be detected by ST, and these are labeled with arrows. The ground truth (green) is also provided. **b** ST-LFV provides tracing results (purple) that are almost equivalent to the ground truth (green). The neurites that cannot be detected with ST can be traced successfully (see the labeled arrow). **c** and **d** Similar to (a) and (b), respectively

Furthermore, we checked that the constructed classifiers (Fig. 10g, h) produce better tracing results. We compared the tracing results from SparseTracer with those given by ST-LFV (Fig. 11a-d), and found that SparseTracer was unable to trace some neurites with weak signals (arrows in Fig. 11a, c). ST-LFV produced tracing results that included almost all of the neurites that could not be traced by SparseTracer. These results verify that the classifiers (Fig. 10g, h) are applicable even when there are several training errors.

**Fig. 12:**
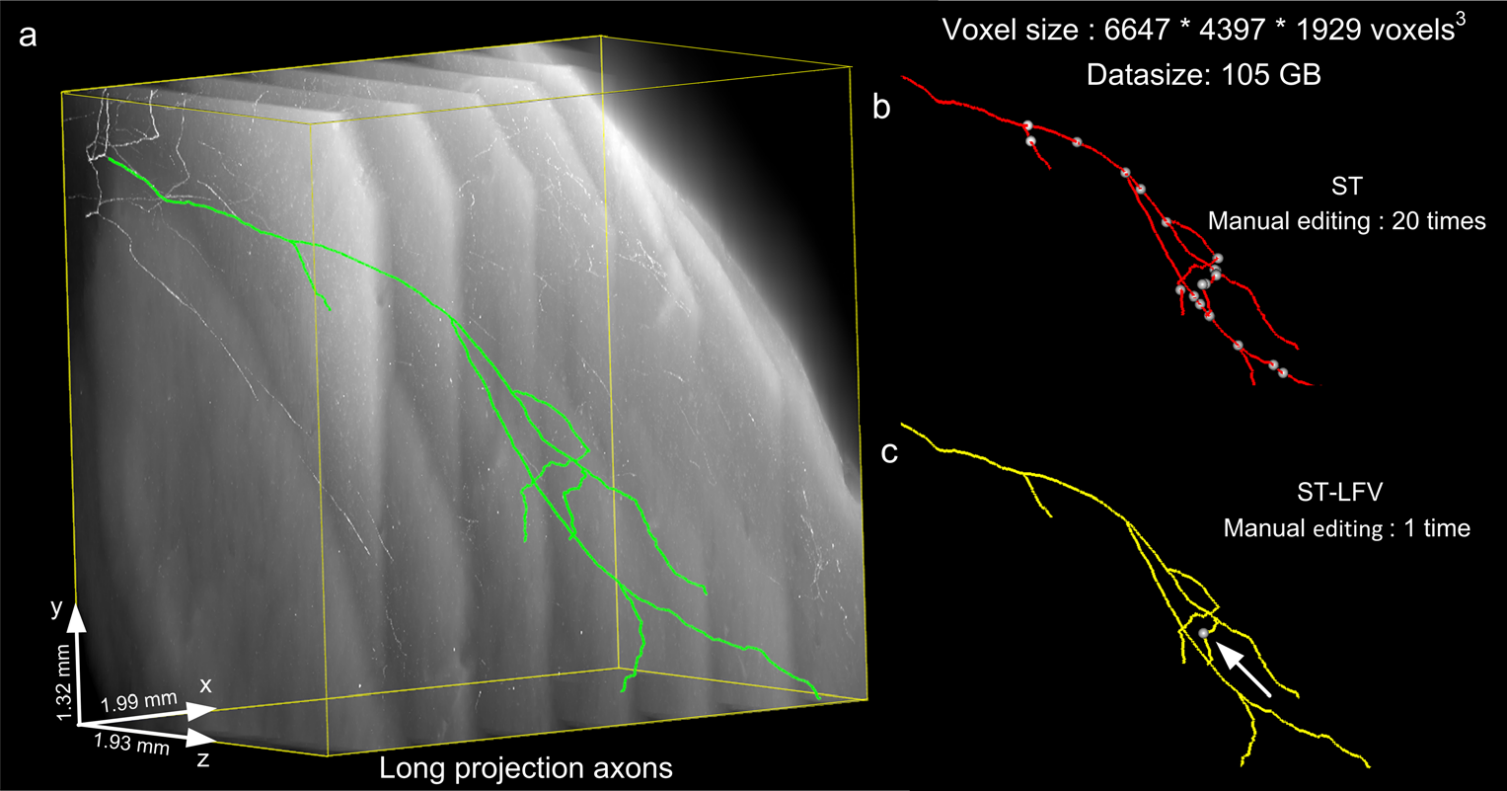
Tracing neurites at a large scale. **a** Large-scale dataset (approximately 105 GB) and the tracing results (ground truth) drawn by a human annotator (green); **b** tracing results (red) drawn by SparseTracer (ST), equivalent to the ground truth. A total of 20 manual edits (interferences) are required, and their corresponding locations are labeled with white dots. **c** For ST-LFV, only one manual edit is required (labeled with an arrow)

The superior tracing performance of ST-LFV resulted in a vast improvement in the automation level of the SparseTracer software, enabling the rapid tracing of neurites in large-scale datasets. We selected a dataset that included several long axons, with a total size of 1.99 × 1.93 × 1.32 mm^3^ (voxel size, 0.3 × 0.3 × 1 μm^3^, 105 GB). We adopted the divide-and-conquer strategy used in our previous work (Li et al. 2016) for the analysis of large datasets. We also added a manual editing module to SparseTracer, to obtain the initial tracing direction and sites for continuous tracing when the weak signal strength fails the detection. This manual editing module allows SparseTracer to produce the same tracing results as ST-LFV (Fig. 12b, c). However, the number of manual editing sessions required for SparseTracer (20 times) is far greater than that for ST-LFV (1 time). This demonstrates the advantage of ST-LFV for large-scale tracing. We quantified this advantage by comparing the total time required while using ST-LFV with that while using SparseTracer. Tracing neurites with SparseTracer requires intensive manual edits, and a skillful annotator may require 20 min. The same annotator would require only 5 min to finish the same task using ST-LFV. In addition, we measured the time required to build the identification model and compared it with that for neurite tracing. Fifteen sub-blocks from the dataset in Fig. 12 were used for this purpose, and the corresponding information is presented in **Table****S3**. According to the tracing pipeline (Method section 2.3), foreground identification is closely linked with neurite tracing and the model only identifies a small part of the failed traced voxels. Therefore, the time required to build and customize the identification model is less than that for tracing neurites. In this comparison, the image reading time was ignored as it would be negligible for the data storage system (a RAID is connected to the workstation directly) used in this study. From the above comparisons, we can conclude that ST-LFV significantly improves the tracing performance of SparseTracer and is a valuable resource for large-scale neurite tracing.

## Discussion

ST-LFV involves more rules deduced from images by human beings than other methods (Li et al. 2017; Chen et al. 2015). These rules are based on several assumptions that are commonly applicable to neuronal images collected with optical microscopes, and provide a basis for constructing a feature vector that displays the differences between foreground and background voxels. The robustness of our model was verified by multi-fold cross validation (see results in **Table****S1**). We also demonstrated that, under our assumptions, the feature vector of a weak signal voxel is essentially different from that of a background voxel (**Fig.****S3**). In our method, a complicated procedure for extracting valid features and identifying weak signals is avoided by using more rules, thus eliminating the need for intensive computation. This is the primary reason why ST-LFV is suitable for large-scale tracing of neurites.

In ST-LFV, unlike other methods (Li et al. 2017; Chen et al. 2015), the identification model is embedded in the tracing procedure. When the tracing termination conditions are triggered, the identification model operates to allow tracing to continue after the model has identified the current tracing point as a foreground voxel. The identification model is linked to the tracing procedure and enhances the ability of ST-LFV to trace neurites with weak signals. Other methods separate the identification and tracing procedures, first using a machine learning method to identify as many foreground voxels as possible and then performing the tracing procedure, i.e., extracting the skeleton based on the identified foreground voxels. These methods aim to identify all foreground voxels, and are therefore relatively computationally expensive, which is an obstacle to the large-scale tracing of neurites. ST-LFV only activates the identification model when the tracing termination conditions have been triggered, and thus only identifies a few foreground voxels when tracing a neurite. This contributes to the ability of ST-LFV to rapidly trace neurites in large-scale images.

Foreground identification is an indispensable step in neurite tracing. The identification model in ST-LFV is used to distinguish foreground voxels from the background. Generally, our identification model could be employed alongside many widely used tracing methods, such as, the model fitting method (Zhao et al. 2011), principal curves method (Bas and Erdogmus 2011), etc. The termination condition of these methods will be activated when the local structure information becomes inadequate. Combined with our model, these methods could potentially identify weaker foreground signals and continue tracing.

As in many machine learning methods (Suykens and Vandewalle 1999; Cortes and Vapnik 1995), obtaining a training set is a key part of ST-LFV. In our method, the training set contains positive (foreground) and negative (background) feature vectors, is automatically generated, and does not require manual labeling. A positive feature vector in the training set is determined by its corresponding foreground point (see the Methods section). The point is automatically generated by the tracing procedure, such as, SparseTracer (Li et al. 2016) or other methods (Bas and Erdogmus 2011; Rodriguez et al. 2009). A negative feature vector is determined by a corresponding point drawn at random (according to a uniform distribution) from the image. This random selection ensures that the chosen point has a very low probability of being in the foreground. The probability is low because the distribution of neurites is sparse and the number of foreground voxels is far smaller than the total number of voxels. According to the above analysis, the automatic generation of a training set from a sparse image is feasible.

In SparseTracer, we used a constrained principal curve to trace neurites with weak signals (Li et al. 2016; Quan et al. 2016). In general, it is difficult to obtain a forward tracing direction from this type of neurite because of inadequate local structure information. In this case, the constrained principal curve introduces directional information for the traced points, and this direction becomes the forward tracing direction. This feature allows SparseTracer to detect weaker signals than other methods (Rodriguez et al. 2009; Wang et al. 2011; Xiao and Peng 2013). When analyzing images that include weak signals, SparseTracer demonstrates highly accurate tracing performance (>85% recall at >90% precision). However, like most other methods, SparseTracer uses a set of thresholds to determine whether the tracing should be terminated. This termination condition may not be suitable when detecting weak signals from an inhomogeneous background (see Figs. 4g, h). This type of weak signal detection is a common task in the process of tracing large-scale neurites. Considering this situation, we proposed an identification model that can be combined with SparseTracer, i.e., ST-LFV, to enable the large-scale tracing of neurites.

The identification model in ST-LFV is based on rules that are suitable for various types of neuronal images. However, directly using the identification model may be inappropriate for images whose characteristics do not satisfy our assumptions. Consider the following two examples. 1) The neurites or somas are densely distributed in the images. In this case, there will be a relatively high number of foreground voxels in the randomly chosen voxels used to construct the negative training set, which will reduce the performance of the identification model. This problem can be addressed by cleaning the negative training set. For instance, the foreground region could be labeled using neurite tracing and soma shape reconstruction methods (Quan et al. 2013; Quan et al. 2014; Luengo-Sanchez et al. 2015; Varando et al. 2018), and then feature vectors whose corresponding voxels were in the labeled regions could be removed from the negative training set. 2) For images without a smooth background, the background features may deviate from the assumption used to construct the identification method. In this case, an anisotropic ROF denoising method (Goldstein and Osher 2009) or bias correction method (Sing et al. 2015) are good approaches to ensure that the smoothed background satisfies our assumption. The selection of the approach shall be based on different image characteristics. In a nutshell, pre-processing methods can extend the application range of our identification model. However, there are some cases that may result in failure to identify weak signals. In case 1), some tracing methods may fail to trace neurites that are highly disconnected, i.e., neurites that can be modeled as a series of sufficiently separable clusters of foreground points rather than as a series of connected cylinders. In this case, neurite tracing failures may result in the unsuccessful application of the identification model. In case 2), there may be images with low z-resolution depths where the neural signal is not sparsely distributed. The identification will then fail because the extraction of background feature vectors will be restricted by the dense foreground voxels.

We demonstrated that ST-LFV can detect weak foreground voxels in tracing sparsely distributed neurites. ST-LFV also has potential advantages in tracing neurites from the large-scale images in which neurites are sparsely distributed. However, it is worth noticing that the identification model in ST-LFV activates only when a starting point and tracing direction are provided. For a partially traced neurite, ST-LFV may generate a complete reconstruction. But it fails in tracing some neurites that are not detected by tracing methods, due to lack of starting traced point and tracing direction. In addition, like other tracing methods (Peng et al. 2010; Peng et al. 2017; Quan et al. 2016; Wang et al. 2011; Wearne et al. 2005), ST-LFV still experiences many difficulties in tracing neurons on a brain-wide scale, which can be attributed to the following causes. First, brain-wide neurite tracing involves identifying individual neurons in the presence of the packed neurites. This problem still challenges the current tracing methods. Second, due to the tree structure of a neuron, the tracing errors can accumulate continuously. Tracing a long-projection neuron makes this situation worse and the tracing errors even extend to the whole brain. This means that for the brain-wide reconstruction, a tracing error will cause an unacceptable tracing result in some cases. Finally, the image stacks with size of terabytes or even tens of terabytes are required to be coped with the brain-wide tracing of neurons, which requires the integration of big data technique and neurite tracing methods. Thus, many challenges still exist in the brain-wide tracing of individual neurons. Aiming to overcome these challenges, appropriate methods should be developed and integrated into a software tool.

## Conclusion

We have proposed a method for identifying weak neurite signals from the background that is inhomogeneous but locally smooth. We verified that the extracted features, which differentiate the foreground and background, are widely applicable to various types of light-microscopic images containing sparsely distributed neurites. The identification method was shown to improve the accuracy of neurite tracing on condition that our rules are consistent with the image characteristics. We further demonstrated that this identification method is suitable for the large-scale tracing of neurites that are sparsely distributed, which may aid in the reconstruction of neurons across different brain regions.

## Information Sharing Statement

Our method is plugged into the software GTree, which is an open source software and available at https://github.com/GTreeSoftware/Release/releases. We also provide some example datasets used in our paper. They can be downloaded at https://github.com/GTreeSoftware/TEST_DATA/releases/tag/ST-LFV. If one is interested in other datasets, please feel free to contact us.

## Electronic supplementary material

ESM 1 (DOCX 1059 kb)
