# Supplementary material for: Identifying Weak Signals in Inhomogeneous Neuronal Images for Large-Scale Tracing of Sparsely Distributed Neurites
Source: Neuroinformatics. 2019 Jan 11;17(4):497–514. doi: 10.1007/s12021-018-9414-9 (PMC6841657; doi:10.1007/s12021-018-9414-9)
Supplement: Supplementary file 1 — (DOCX 1059 kb) [file 12021_2018_9414_MOESM1_ESM.docx]

**Supplementary figures**

**
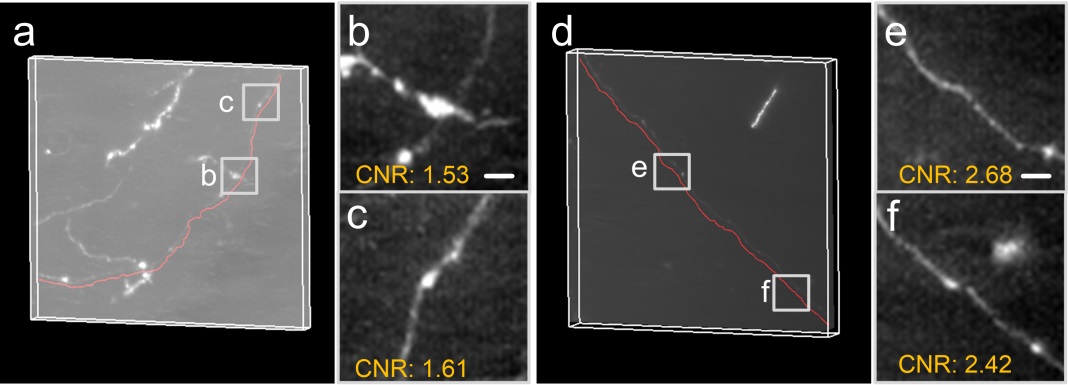
**

**Figure S1.** CNR values of sub-blocks in datasets in Fig. 4. (a) One dataset with a manually traced neurite (red). Two areas which contain a part of traced neurites are labeled. The size of sub-blocks (b) & (c) are 50 × 50 × 15 voxels. The maximum projections of these two regions through a depth of 15 μm, with a scale bar of 10 μm; the computed CNR values of the sub-blocks are highlighted in the figure. (d) One dataset with a weak background and others are similar to (a); The size of sub-blocks (e) & (f) are 50 × 50 × 30 voxels, (e) and (f) have similar descriptions as (b) and (c), respectively.


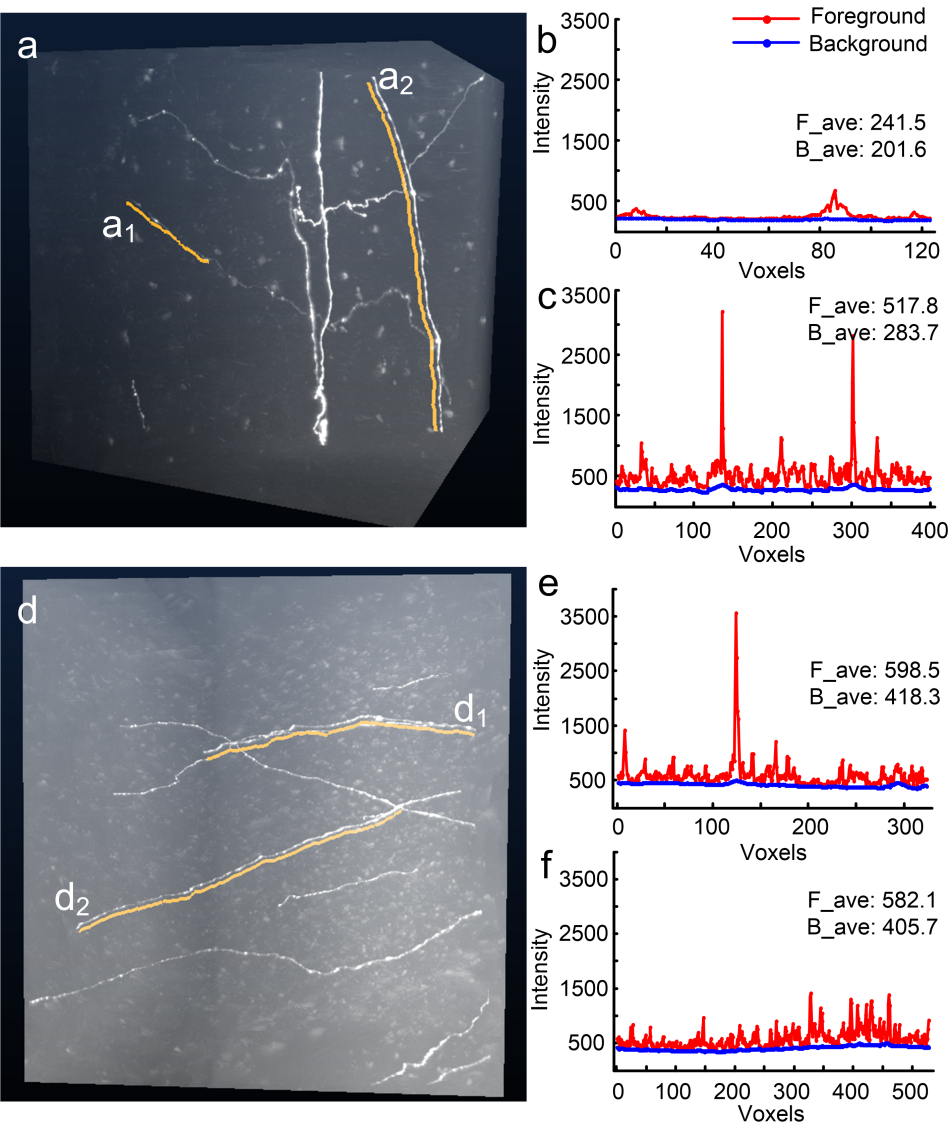


**Figure S2**. Foreground and estimated background intensities of the manually traced neurites. (**a**) is a dataset used in Fig. 6 (data 11); **a_1_** and **a_2_** are two manually traced skeletons (yellow) shown in the stack. The traced skeletons are slightly shifted from the original data; (**b**) - (**c**) illustrate the foreground and estimated background intensities of the traced skeletons a_1_ and a_2_, respectively. The average intensity of foreground and background are computed and shown with “F_ave” and “B_ave”; (**d**) is another dataset used in Fig. 6 (data 5); **d_1_** and **d_2_** are the manually traced skeletons; (**e**) - (**f**) Foreground (red) and background intensities (blue) of the traced skeletons in (**d**), the average intensity of foreground and background are also given in these sub-figures.


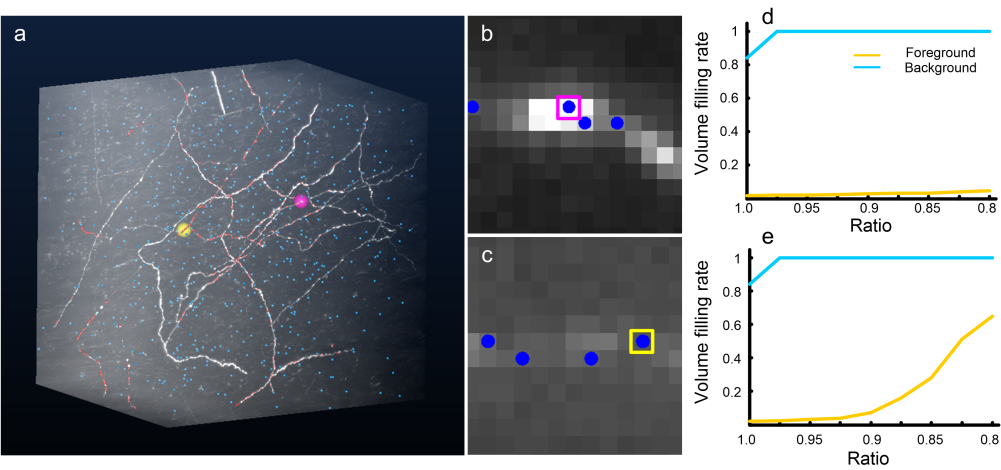


**Figure S3.** The feature vectors of foreground voxels with strong and weak signal intensities. (**a**) An image stack including many neurites. The red and blue dots label the voxels for constructing positive and negative training sets, respectively Two foreground voxels with strong (purple) and weak (yellow) signal intensities are selected respectively. (**b**) & (**c**) are 2D view of the two selected voxels dots in (**a**) and their neighboring image region., Their locations are highlighted with squares in same color of the balls in (**a**); (**d**) the feature vector of the strong signal voxel highlighted in (**b**) is plotted (yellow), as well as the feature vector of a background voxel; (**e**) the feature vector of the weak signal voxel highlighted in (**c**), other descriptions are similar with (**d**).

**Supplementary note**

In the manuscript, we used contrast-to-noise ratio, CNR to evaluate the identifying ability of our method. The CNR calculation is listed below.

1. The experimental datasets in our manuscript are filtered images which acquired by light microcopy. The exact noise level of these images is scarcely possible being evaluated. Thus, we compute the image background instead of noise, and use the following equation to evaluate CNR of some target areas ([Song et al. 2004](#_ENREF_3)),

$CNR_{1}\text{=}\frac{\text{contrast}}{\text{noise}}\text{=}\frac{\Delta_{\mu}}{\text{noise}}\text{=}\frac{\mu_{ROI}-\mu_{mean}}{[(\sigma_{ROI}^{2}+\sigma_{mean}^{2})/2]^{1/2}}$ (Eq. 1)

where μ_ROI_ is the mean of the voxel intensities in the target area; μ_mean_ is the mean value of the averaged intensities in the total ROI-shaped background areas; the background area has the same size as the target area. σ_ROI_ and σ_mean_ are the standard deviations of the target and the background areas, respectively.

We follow the following steps to compute the CNR value of a target neurite.

Step1) consider the neurite in the image as a target ROI, reconstruct the shape of the neurites, and label the voxels in the reconstructed shape.

Step2) estimate a background image which has the same size as original image (Quan et al. 2013; Quan et al. 2014) and label the voxels whose coordinates have the same as that of labeled voxels in step 1).

Step3) computed the mean of intensities of all the labelled voxels on the original image and the estimated background image, which lead to *μ_ROI_* and μ_mean_. The computing of standard deviation values is similar with mean values. Finally, a CNR value is computed follow the Eq. 1.

1. The noise level of synthetic datasets is predetermined. Here, the CNR measurement is given by ([Welvaert and Rosseel 2013](#_ENREF_4)).

$\frac{A}{\sigma_{N}}$ (Eq. 2)

Where A is the amplitude of the signal, equal to the signal peak subtracting to the background.

**Supplementary Tables**

| Table S1. The estimate error with multi-fold cross-validation | |
| --- | --- |
| Data id  (source) | Cross-validated estimate error (%) |
| 1 (data 1 in Fig. 6) | 0.15 |
| 2 (data 2 in Fig. 6) | 2.10 |
| 3 (data 3 in Fig. 6) | 0.15 |
| 4 (data 4 in Fig. 6) | 0.05 |
| 5 (data 5 in Fig. 6)  6(data 6 in Fig. 6)  7 (data 7 in Fig. 6)  8 (data 8 in Fig. 6)  9 (data 9 in Fig. 6)  10 (data 10 in Fig. 6)  11 (data 11 in Fig. 6)  12 (data 12 in Fig. 6)  13 (Fig. 9a)  14 (Fig. 9b)  15 (Fig. 10a)  16 (Fig. 10b) | 0.10  0.30  0.50  0.40  0.40  0.40  0  0.10  0.75  0.05  0.05  0.10 |

| Table S2 Comparisons on SparseTracer, ST-LFV, UltraTracer and Open-Snake in tracing accuracy on various datasets | | | | | | | | | |
| --- | --- | --- | --- | --- | --- | --- | --- | --- | --- |
| Data ID | Data source reference | Precision/Recall (%) | | | | | | | |
|  |  | SparseTracer | | ST-LFV | | UltraTracer | | Open-Snake | |
| a | Peng et al., 2015 | 99 | 99 | 94 | 99 | 99 | 94 | 90 | 99 |
| b | Peng et al., 2017 | 99 | 92 | 99 | 94 | 99 | 92 | 93 | 75 |
| c | Peng et al., 2015 | 97 | 94 | 93 | 95 | 97 | 68 | 95 | 82 |
| d | Brown et al., 2011 | 82 | 96 | 90 | 96 | 91 | 97 | 62 | 95 |
| e | Gong et al., 2013 | 99 | 82 | 99 | 97 | 74 | 47 | 95 | 66 |
| f | Gong et al., 2013 | 98 | 45 | 95 | 100 | N/A | N/A | N/A | N/A |
| The best values are labeled with a bold letter | | | | | | | | | |

| Table S3. The neurite length, time cost, speed and throughput of tracing sub-blocks with ST-LFV at large-scale | | | | | |
| --- | --- | --- | --- | --- | --- |
| ID | Length (μm) | Time cost (ms) | | Speed (μm/ms) | Throughput  (×10^3^ voxel^3^ / ms) |
|  |  | Identification* | Tracing** | Length / Time cost | Size*** / Time cost |
| 1 | 58.7 | 402 | 298 | 0.08 | 2.28 |
| 2 | 47.0 | 179 | 201 | 0.12 | 4.20 |
| 3 | 73.1 | 218 | 216 | 0.17 | 3.68 |
| 4 | 55.4 | 293 | 297 | 0.09 | 2.70 |
| 5 | 78.4 | 260 | 218 | 0.16 | 3.34 |
| 6 | 54.1 | 277 | 302 | 0.09 | 2.76 |
| 7 | 72.0 | 230 | 262 | 0.15 | 3.24 |
| 8 | 44.4 | 143 | 201 | 0.13 | 4.64 |
| 9 | 70.2 | 145 | 217 | 0.19 | 4.41 |
| 10 | 50.2 | 156 | 210 | 0.14 | 4.36 |
| 11 | 49.5 | 151 | 224 | 0.13 | 4.26 |
| 12 | 46.9 | 145 | 230 | 0.13 | 4.26 |
| 13 | 48.1 | 168 | 215 | 0.13 | 4.17 |
| 14 | 48.1 | 192 | 264 | 0.11 | 3.50 |
| 15 | 35.9 | 208 | 286 | 0.07 | 3.23 |
| **Average** | **55.47** | **211.13** | **242.73** | **0.13** | **3.67** |
| Identification* indicates building and customizing SVM model. Tracing** indicates trace the neurites in the sub-block. Size***: the voxel size of each sub-block is 121×121×109 voxel^3^. | | | | | |

**Reference**

Brown, K. M., Barrionuevo, G., Canty, A. J., De Paola, V., Hirsch, J. A., Jefferis, G. S., et al. (2011). The DIADEM data sets: representative light microscopy images of neuronal morphology to advance automation of digital reconstructions. *Neuroinformatics, 9*(2-3), 143-157.

Gong, H., Zeng, S., Yan, C., Lv, X., Yang, Z., Xu, T., et al. (2013). Continuously tracing brain-wide long-distance axonal projections in mice at a one-micron voxel resolution. *Neuroimage, 74*, 87-98.

Peng, H., Hawrylycz, M., Roskams, J., Hill, S., Spruston, N., Meijering, E., et al. (2015). BigNeuron: large-scale 3D neuron reconstruction from optical microscopy images. *Neuron, 87*(2), 252-256.

Peng, H., Zhou, Z., Meijering, E., Zhao, T., Ascoli, G. A., & Hawrylycz, M. (2017). Automatic tracing of ultra-volumes of neuronal images.*Nat. Methods, 14*(4), 332.

Quan, T., Li, J., Zhou, H., Li, S., Zheng, T., Yang, Z., et al. (2014). Digital reconstruction of the cell body in dense neural circuits using a spherical-coordinated variational model. *Sci. Rep., 4*, 4970.

Quan, T., Zheng, T., Yang, Z., Ding, W., Li, S., Li, J., et al. (2013). NeuroGPS: automated localization of neurons for brain circuits using L1 minimization model. *Sci. Rep., 3*.

Song, X., Pogue, B. W., Jiang, S., Doyley, M. M., Dehghani, H., Tosteson, T. D., et al. (2004). Automated region detection based on the contrast-to-noise ratio in near-infrared tomography. *Applied Optics, 43*(5), 1053-1062.

Welvaert, M., & Rosseel, Y. (2013). On the definition of signal-to-noise ratio and contrast-to-noise ratio for fMRI data. *PLoS One, 8*(11), e77089.
